# In Vivo Metabolite Profiling of DMU-212 in Apc^Min/+^ Mice Using UHPLC-Q/Orbitrap/LTQ MS

**DOI:** 10.3390/molecules28093828

**Published:** 2023-04-30

**Authors:** Jing Li, Xinghua Li, Xiaohang Zhou, Le Yang, Hui Sun, Ling Kong, Guangli Yan, Ying Han, Xijun Wang

**Affiliations:** 1 National Chinmedomics Research Center, National TCM Key Laboratory of Serum Pharmacochemistry, Metabolomics Laboratory, Department of Pharmaceutical Analysis, Heilongjiang University of Chinese Medicine, Heping Road 24, Harbin 150040, China; 2 State Key Laboratory of Quality Research in Chinese Medicine, Macau University of Science and Technology, Avenida Wai Long, Taipa, Macau 999078, China; 3 State Key Laboratory of Dampness Syndrome, The Second Affiliated Hospital Guangzhou University of Chinese Medicine, Dade Road 111, Guangzhou 510006, China

**Keywords:** DMU-212, colorectal adenoma, UHPLC-Q/Orbitrap/LTQ MS, metabolites

## Abstract

3,4,5,4’-Trans-tetramethoxystilbene (Synonyms: DMU-212) is a resveratrol analogue with stronger antiproliferative activity and more bioavailability. However, the metabolite characterization of this component remains insufficient. An efficient strategy was proposed for the comprehensive in vivo metabolite profiling of DMU-212 after oral administration in Apc^Min/+^ mice based on the effectiveness of the medicine. Ultra-high performance liquid chromatography-quadrupole/orbitrap/linear ion trap mass spectrometry (UHPLC-Q/Orbitrap/LTQ MS) in the AcquireX^TM^ intelligent data acquisition mode, combining the exact mass and structural information, was established for the profiling and identification of the metabolites of DMU-212 in vivo, and the possible metabolic pathways were subsequently proposed after the oral dose of 240mg/kg for 3 weeks in the colorectal adenoma (CRA) spontaneous model Apc^Min/+^ mice. A total of 63 metabolites of DMU-212 were tentatively identified, including 48, 48, 34 and 28 metabolites in the Apc^Min/+^ mice’s intestinal contents, liver, serum, and colorectal tissues, respectively. The metabolic pathways, including demethylation, oxidation, desaturation, methylation, acetylation, glucuronide and cysteine conjugation were involved in the metabolism. Additionally, further verification of the representative active metabolites was employed using molecular docking analysis. This study provides important information for the further investigation of the active constituents of DMU-212 and its action mechanisms for CRA prevention.

## 1. Introduction

Colorectal cancer (CRC) is a malignant tumor of the gastrointestinal tract that is highly likely to metastasize and recur, and it is associated with an extremely high mortality rate [1]. Colorectal adenoma (CRA) is a frequent intestinal mucosal disease, which can progress into CRC in the adenoma-carcinoma sequence [2]. Early chemopreventive interventions of precancerous CRA are the most effective measure for the reduction in mortality and morbidity associated with CRC [3]. Currently, nonsteroidal anti-inflammatory drugs, COX-2 inhibitors, and calcium are potential candidates as chemopreventive agents for CRC. However, the limitation of a lower effectiveness and side effects hindered further application [4].

**Figure 1:**
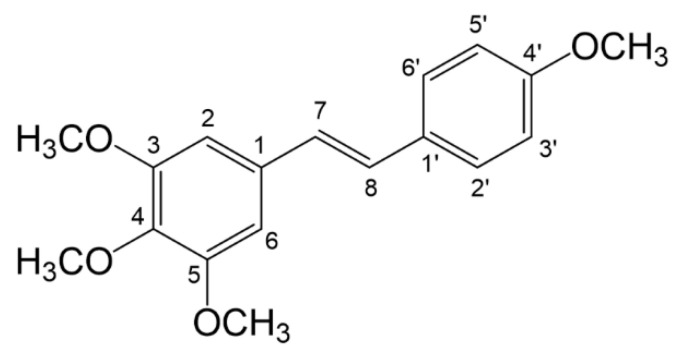
The chemical structure and numbering of DMU-212.

3,4,5,4’-Trans-tetramethoxystilbene (Synonyms: DMU-212, Figure 1) is a methylated analogue of resveratrol possessing greater bioavailability and stronger anti-cancer activity [5]. It has been examined as a potential chemopreventive agent that can prevent CRC progression through reducing the number of adenomas and suppressing tumor formation in the colon of the Apc^Min/+^ mice; the inhibitory effect of DMU-212 on CRA growth may be related to the interference with prostaglandin E-2 generation in cells [6]. In a previous study of ours, we demonstrated that the mechanisms of CRA prevention using DMU-212 were related to gut microbiota and its metabolites (unpublished data) and, thus, DMU-212 has the potential for use as a chemopreventive agent to prevent CRC. Despite the outstanding efficacy of DMU-212 for anti-CRA, the active constituents and in vivo metabolism study of DMU-212 related to the pharmacological effect are uncertain.

According to one study, DMU-212 undergoes metabolic oxidation, hydroxylation and O-demethylation reactions in liver extracts of mice and an incubate of mouse liver microsomes, as a result of which five metabolites are generated: DMU-214, DMU-281, DMU-291, DMU-295 and DMU-807. DMU-212, administrated to mice, produced higher levels of drug in the small intestine, colon mucosae, and brain than resveratrol. Furthermore, DMU-212 is more easily able to cross the blood-brain barrier than resveratrol due to its higher lipophilicity [7]. It is of utmost importance to identify the metabolites and metabolic pathways of a drug during drug discovery and development. However, taking into highly complex biological matrices, discovering possible metabolites, and identifying the significant features remains a big challenge [8]. Only a few corresponding metabolites of DMU-212 have been identified using the Quattro Bio-Q tandem quadrupole mass spectrometer upgraded to the Quattro MK II specifications in healthy mice [7]. This was not sufficient for the research on the active constituents and action mechanisms of DMU-212 for anti-CRA. Consequently, it is necessary to develop efficient strategies that could identify and targeted detect the numerous trace-absorbed prototypes and metabolites from the complex biological matrix to systematically identify and characterize the absorbed components of DMU-212 in CRA spontaneous model Apc^Min/+^ mice.

The Orbitrap ID-X Tribrid mass spectrometer with a quadrupole, orbitrap analyzer, and linear ion trap has an enhanced mass resolution power, scan speed, and the ability to perform MS^n^ for better structural characterization. The AcquireX^TM^ data acquisition workflow includes an automatic background subtraction, fast polarity-switching mode, isotope in-tensity-filtering, and other real-time decision-making features. Compound Discoverer 3.3 software carried out the metabolite analysis and compound annotation. Thus, the chemical constituents in complex matrix samples can be detected at trace levels in a sensitive and high-resolution platform. In the present study, a comprehensive strategy was proposed for the rapid identification of the metabolites of DMU-212 after the oral dose of 240 mg/kg for 3 weeks in CRA spontaneous model Apc^Min/+^ mice based on ultra-high performance liquid chromatography-quadrupole/orbitrap/linear ion trap mass spectrometry (UHPLC-Q/Orbitrap/LTQ MS) combined with the data processing software “Compound Discoverer 3.3” and the AcquireX^TM^ data acquisition workflow. The representative active metabolites were supported by data from molecular docking assays. This study provides valuable information regarding DMU-212’s active constituents and its mechanism of action for preventing CRA.

## 2. Results

### 2.1. Establishment of the Analytical Strategy

**Figure 2:**
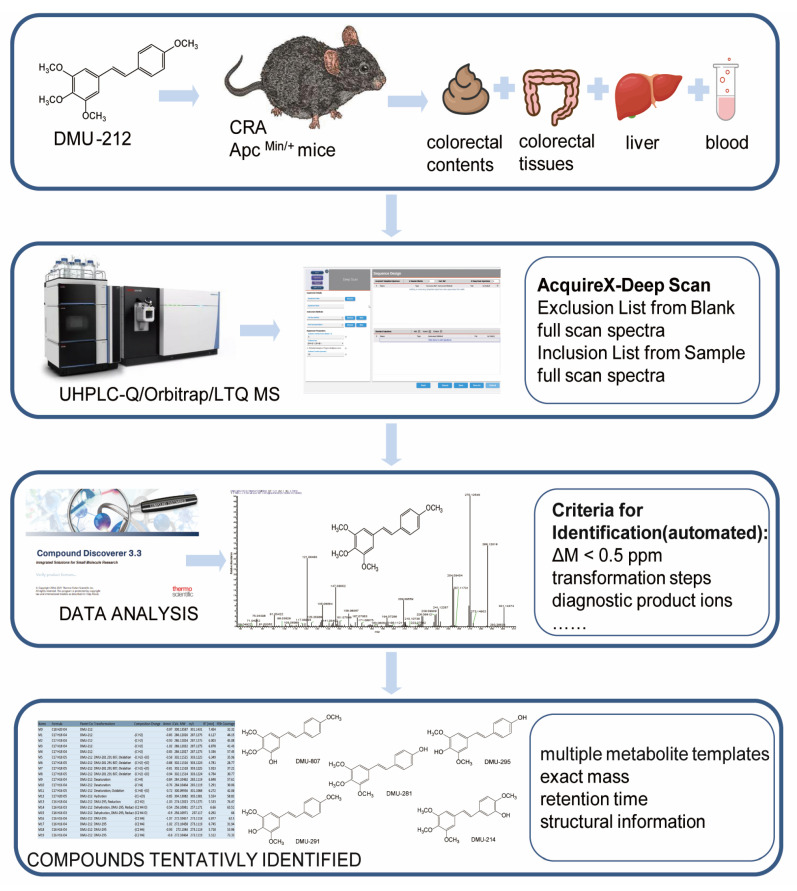
The summary analytical strategy diagram for the detection and identification of DMU-212 metabolites.

In this paper, an effective strategy was established for metabolite identification of DMU-212 in the Apc^Min/+^ mice’s blood, liver, colorectal tissues and intestinal contents using UHPLC-Q/Orbitrap/LTQ MS coupled with multiple data-processing methods. Firstly, data extraction was used to obtain and interpret the data from the normal and CRA samples using qualitative Acquire X^TM^ iterative data dependent MS^2^ (dd-MS^2^) and quantitative full-MS analyses. The inclusion and exclusion lists are automatically generated in Deep Scan mode upon the injection of the blank and sample, respectively. In the iterative injections of the drug-containing sample, both lists (inclusion/exclusion) are automatically updated, allowing a comprehensive acquisition of the relevant features, excluding those present in the blanks. Then, the raw control and sample files are imported into the Compound Discovery software, and the DMU-212 structure was analyzed to acquire the cleavage pathways and diagnostic product ions for metabolite identification. The workflow parameters include the type of transformation steps and list of possible adduct ions, and it also performs a retention time alignment, unknown compound detection, and compound grouping. An expected ion list was generated based on the above. Thirdly, the metabolite templates were summarized and established using the reported metabolic transformations of the ingredients in the literature. Compared with the blank biosamples, including blood, liver, colorectal tissues and intestinal contents, the DMU-212 metabolites in the biosamples were identified using multiple metabolite templates, the exact mass, retention time, and structural information. The summary analytical strategy diagram is shown in Figure 2.

### 2.2. Characteristic Fragments of DMU-212

**Figure 3:**
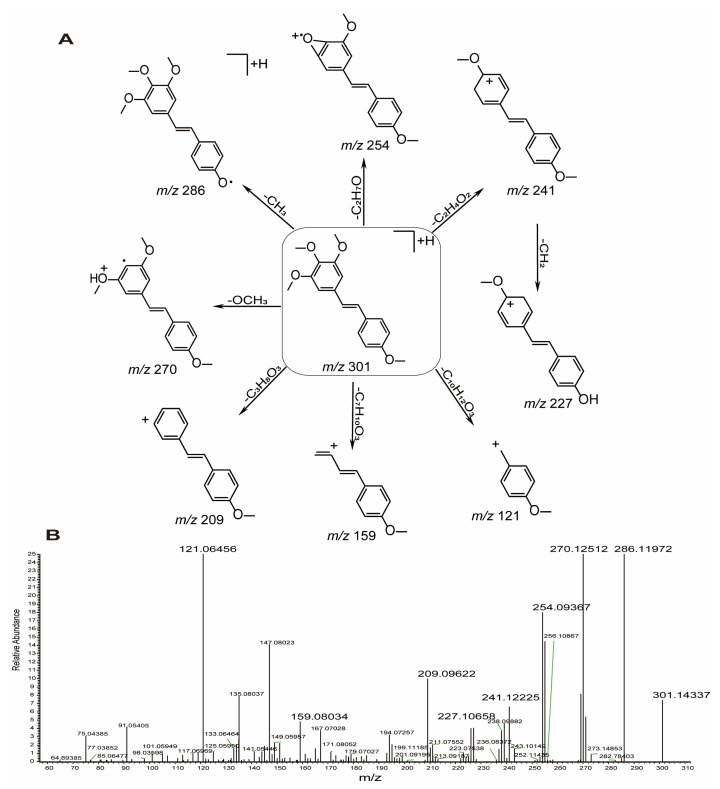
Mass fragmentation behavior (**A**) and the MS^2^ spectrum (**B**) of DMU-212 in positive ion modes.

DMU-212 generated a quasi-molecular ion [M + H]^+^ ion at *m*/*z* 301.14,337 (C_18_H_21_O_4_, error 0.23ppm). In the MS/MS spectra, *m*/*z* 301 ions lost the methyl group at the 4’-carbon position to produce ions at *m*/*z* 286 [M + H-CH_3_]^+^, or removed the methoxy group at the 4-carbon position to produce ions at *m*/*z* 270 [M + H-OCH_3_]^+^. Fragment ions at *m*/*z* 254 [M + H-OCH_3_-CH_4_]^+^ are the fragment ions corresponding to the loss of methyl and methoxy results in ring-closing at the 4, 5-carbon position from the [M + H]^+^ ions of DMU-212. The product ion at *m*/*z* 241 [M + H-2OCH_2_]^+^ is formed by the loss of two OCH_2_ molecules at the 3, 5-carbon position from the precursor ion, which further releases a CH_2_ molecule at the 4’-carbon position to form ions at *m*/*z* 227 [M + H-2OCH_2_-CH_2_]^+^. The fragment ion at *m*/*z* 209 [M + H-2OCH_3_-OCH_2_]^+^ corresponds to the [M + H]^+^ quasi-molecular ion with the loss of one OCH_2_ molecule and two methoxy groups. The ion at *m*/*z* 159 [M + H-C_7_H_10_O_3_]^+^ is formed by the benzene ring breakage between C-1, 6 and C-2, 3 and the ion at *m*/*z* 121 is formed by the double bond breakage between C-7 and C-8 from the [M + H]^+^ quasi-molecular ion. Based on the above analysis, characteristic fragment ions including *m*/*z* 301, *m*/*z* 286, *m*/*z* 270, *m*/*z* 254, *m*/*z* 241, *m*/*z* 227, *m*/*z* 209, *m*/*z* 159 and *m*/*z* 121 were serving as characteristic product ions for the identification of the DMU-212 metabolites. The proposed fragmentation pathways of DMU-212 and the MS^2^ spectrum are shown in Figure 3.

### 2.3. Comprehensive Characterization of Metabolites of DMU-212 In Vivo 

After intragastric gavage (i.g.) administration to the Apc^Min/+^ mice, DMU-212 (M0) was detected in the blood, liver, colorectal tissues and intestinal contents samples, according to the retention time (t R = 7.454 min), the accurate quasimolecular ion, as well as the characteristic product ions. DMU-212 was detected in all samples in vivo. A total of 63 DMU-212 metabolites were detected and characterized from the Apc^Min/+^ mice’s blood, liver, colorectal tissues and intestinal contents samples according to the suggested transformation pathways, molecular formula, and mass spectrometry fragment ions by means of the UHPLC-Q/Orbitrap/LTQ MS method coupled with the established strategy. All the information on the 63 metabolites of DMU-212 detected using UHPLC-Q/Orbitrap/LTQ MS are listed in Table S2. In addition, Supplementary Figure S4 shows the MS/MS spectrum of DMU-212 and its metabolite.

#### 2.3.1. Phase I Metabolites Identification

Demethylated and/or desaturated metabolites:

The molecular weights of metabolites M1-4, M16-22, M29 and M30 were all multiples of 14 Da (CH_2_) less than that of DMU-212, which indicated the loss of the methyl chain moiety, and the characteristic ions were in accordance with the speculative structure. Metabolites M34–36 showed the [M + H]^+^ ions at around *m*/*z* 299 (C_18_H_18_O_4_). Their molecular weights were all 2 Da (2H) less than that of the DMU-212, so this metabolite showed the characteristic fragments with *m*/*z* 254, 241, 209 and 121, indicating that they are the desaturated metabolites of DMU-212. Metabolites M9 and M10 showed the [M + H]^+^ ion at *m*/*z* 285 (C_17_H_16_O_4_) with a mass shift of 16 Da, with just the loss of one CH_2_(14 Da) and one H_2_ (2 Da) group. In the MS^2^ spectra, the characteristic fragment ions showed the ion at *m*/*z* 270, 254, 241, 227, 209, 159 and 121. Similarly, the molecular weights of metabolites M25 and M26 were all 30 Da less than that of DMU-212, this mass difference corresponds to the loss of the 2H and 2CH_2_ groups. In addition, the difference of M32 in mass (300 Da versus 256 Da) corresponds to the loss of the 2H and 3CH_2_ groups, indicating that they were all the desaturated (loss of 2 H) and demethylated metabolites of DMU-212.

Oxidized/Oxidized and desaturated metabolites:

Metabolites M41-45, the oxidation products in the benzene ring, gave the [M + H]^+^ ions at around *m*/*z* 317 (C_18_H_20_O_5_), which is 16 Da (O) more than that of DMU-212, and the characteristic ions were in accordance with the speculative structure. Metabolites M39-40 (*m*/*z* 313) were the oxidized products in the benzene ring, with the four H atoms leaving. Metabolites M38 showed the [M + H]^+^ ions at around *m*/*z* 331 (C_18_H_18_O_6_), which is 30 Da (O) more than that of DMU-212. The difference in mass corresponds to the binding of two oxygen atoms and the loss of two hydrogen atoms. In the MS 2 spectra, these metabolites had common fragments with the parent drug, indicating they are the oxidized and desaturated derivatives of DMU-212.

Demethylated and dehydrated (and reduced/desaturated) metabolites:

Metabolites M23 and M24 were detected at the retention time of 6.331 and 6.144 min, respectively. They both showed the [M + H]^+^ ions at around *m*/*z* 255 (C_16_H_14_O_3_), which is 56 Da (C_2_H_6_O) less than that of DMU-212. This mass difference corresponds to the loss of one water molecule and two CH_2_ groups. In addition, the fitted chemical formula of M31 was C_15_H_12_O_3_, a difference of C3H8O with DMU-212, which indicated the loss of one water molecule and three CH_2_ groups. Furthermore, metabolites M14-15 generated a protonated [M+ H]^+^ ion at *m*/*z* 257 with the predictive molecular formula of C_16_H_16_O_3_. DMU-212 yielded a difference of C_4_H_8_O with the *m*/*z* 257 by successively losing H_2_O and two CH_2_ (14Da). Metabolites M14-15 further lost four H atoms via a desaturation reaction to form metabolites M27 with *m*/*z* 253 (C_16_H_12_O_3_). The characteristic ions were in accordance with the above speculative structure, suggesting that these metabolites could be the demethylated and dehydrated (and reduced/desaturated) metabolites of DMU-212.

Demethylated and oxidized/reduced metabolites:

Metabolites M5-8 were, respectively, eluted at 6.349, 4.781, 5.923, and 6.784 min and all exhibited a protonated molecule ion at *m*/*z* 303(C_17_H_18_O_5_), 2 Da more than that of DMU-212 (-CH_2_+O), suggesting that it is possibly an oxidized and demethylated product of DMU-212. Metabolites M13 and M28, with [M + H]^+^ ions at around *m*/*z* 275 and 261, were eluted at 5.533 and 5.743 min, respectively. They were 26 Da or 40 Da less than DMU-212 with the formula C_16_H_18_O_4_ or C_15_H_16_O_4_, suggesting that it lost two or three methyl moieties and added two hydrogen atoms. All these metabolites had common fragments with the parent drug.

Other Phase I metabolites:

The fitted chemical formula of M11 was C_17_ H_16_ O_5_, with [M + H]^+^ ions at around *m*/*z* 301, a difference of (-CH_4_+O) with DMU-212, which indicated the loss of the alkyl chain moiety, oxidation in the benzene ring and desaturation, and the characteristic ions were in accordance with the speculative structure. Metabolite M12, with [M + H]^+^ ions at around *m*/*z* 305, was eluted at 5.554 min. The metabolite was 4 Da more than DMU-212 with the formula C_17_H_20_O_5_, which was yielded via demethylation of the alkyl chain and hydration, and the characteristic fragment ions at the MS^2^ spectra, such as *m*/*z* 241, 121 were able to be detected. Metabolite M59 was found at 5.846 min with [M + H]^+^ ions at around *m*/*z* 319 (with a mass shift of 18 Da, exactly bound to a water molecule), so this metabolite showed the characteristic fragments and could be a hydrated metabolite of DMU-212.

#### 2.3.2. Phase II Metabolites Identification

Glucuronide-conjugated and/or methylated metabolites:

Metabolite M56 was eluted at 6.112 min and exhibited a molecular formula of C_23_H_26_O_10_ (*m*/*z* 463 [M + H]^+^). The molecular weight of M56 was 162 Da more than the parent drug, it was a glucuronide conjugate, obviously. The fitted molecular formula for metabolite M57 was C_23_H_26_O_11_ (*m*/*z* 479 [M + H]^+^), indicating that oxidation happened on the basis of metabolite M35. Metabolites M47 (t R = 5.257 min) and M48 (t R = 8.077 min) have the same molecular formula of C_19_H_22_O_4_, deduced from their [M + H]^+^ ions at *m*/*z* 315. In addition, compared with the parent drug, M47 and M48 may bind to a CH_2_ group to form methylated metabolites. Metabolite M51–53 were, respectively, detected at 7.460, 8.936 and 7.912 min with the mass charge ratio 313. Furthermore, the fitted molecular formula was C_19_H_20_O_4_, just a difference of 2H with the M47 and M48. Accordingly, they were tentatively identified as the loss of 2H and a methylated product of DMU-212. Metabolite M58 generated the [M + H]^+^ ion at *m*/*z* 477 with the molecular formula C_24_H_28_O_10_, and its retention time was 6.302 min, a difference of C_6_H_8_O_6_ with DMU-212, which indicated the conjugation of glucuronide (C_5_H_6_O_6_) and a methyl chain moiety (CH_2_). Further, the characteristic ions were in accordance with the secondary fragments of the above metabolite.

Acetylated (and methylated) metabolites:

Metabolite M49 was detected at 5.873 min. It generated the protonated molecule ion at around *m*/*z* 329 (C_19_H_20_O_5_), 28 Da more than that of DMU-212 (+CO), suggesting that it was an acetylated product of DMU-212. Metabolite M50 was eluted at 6.599 min, which exhibited the molecular formula of C_19_H_20_O_6_ ([M + H]^+^, *m*/*z* 345), indicating that oxidation happened on the basis of M49. Metabolite M37 was speculated to be C_19_H_18_O_5_ according to the spectral data. It was 26 Da (+CO − H2) higher than that of DMU-212 and was deduced as an acetylated and desaturated metabolite of DMU-212. The fitted molecular formula for metabolite M60 (*m*/*z* 345 [M + H]^+^) was C_20_H_24_O_5_, this suggests that acetylation and methylation may have occurred on the basis of the reduced DMU-212. Importantly, the fragment ions of the above metabolites are consistent with the characteristic fragments of DMU-212.

Cysteine-conjugated metabolites:

M54 and M55 were eluted at 5.442 and 5.532 min, respectively. The two metabolites possessed the common molecular formula of C_21_ H_27_ N O_7_ S with the experimental [M + H]^+^ ions at *m*/*z* 438. Comparing their molecular formula with that of M0, it is presumed that they might undergo oxidation and combine with cysteine. Metabolites M62 and M63 were eluted at 5.564 and 5.647 min, respectively. Two of them gave the same theoretical mass (*m*/*z* 436) and formula (C_21_H_25_NO_7_S). They were 2 Da (H_2_) more massive than M54–55, indicating that they might undergo oxidation and combine with cysteine on the basis of desaturated DMU-212. Metabolite M46 was eluted at 3.867 min with the [M + H]^+^ ion at *m*/*z* 392 (C_19_H_21_NO_6_S). As it was 91 Da more massive than DMU-212, and the difference in mass corresponds to the loss of two CH_2_ groups and four hydrogen atoms, and its combining with cysteine, it was speculated to be a demethylated, desaturated and cysteine-conjugated product of DMU-212. The fragment ions of the above metabolites match the characteristic fragments of DMU-212, which is an important part of metabolite characterization.

Glycine or glutamine-conjugated metabolites:

M33 eluted at 6.325 min with a [M + H]^+^ ion at *m*/*z* 330 (C_18_H_19_NO_5_), which was 29 Da (-H+NO) larger than DMU-212; its fragments were consistent with those of DMU-212, suggesting that M33 was a glycine conjugate. Metabolites M61, with [M + H]^+^ ions at *m*/*z* 399 (C_21_H_22_N_2_O_6_), were eluted at 5.002 min. A mass increase of 98 D corresponds to the loss of two CH_2_ groups, one oxygen atom and four hydrogen atoms, and its combining with glutamine. The fragments ions of M61 also coincide with the characteristic fragments of DMU-212.

### 2.4. Metabolic Profiles of DMU-212

**Figure 4:**
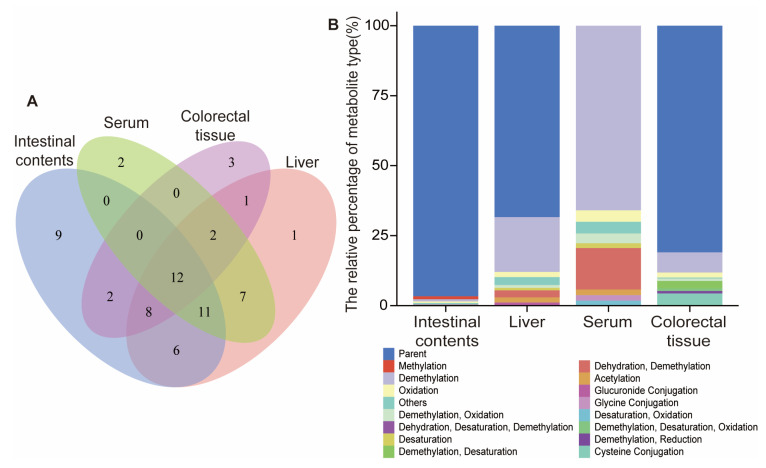
The numbers of metabolites of DMU-212 in each sample (**A**) and the main metabolite types (**B**).

A total of 63 metabolites with different structures were observed and identified in the biosamples in vivo (Figure 4A). Among them, 34 metabolites were detected in the Apc^Min/+^ mice’s serum and all of them can be observed in the Apc^Min/+^ mice’s liver (48 metabolites) except for two unique metabolites. Another three unique metabolites were also identified in the Apc^Min/+^ mice’s colorectal tissues and the rest of the 25 metabolites in colorectal tissues can be found in the serum, liver or intestinal contents, of which 2 metabolites can only be detected in the intestinal contents (50 metabolites) and 1 metabolite can only be obtained in the livers of the Apc^Min/+^ mice.

**Figure 5:**
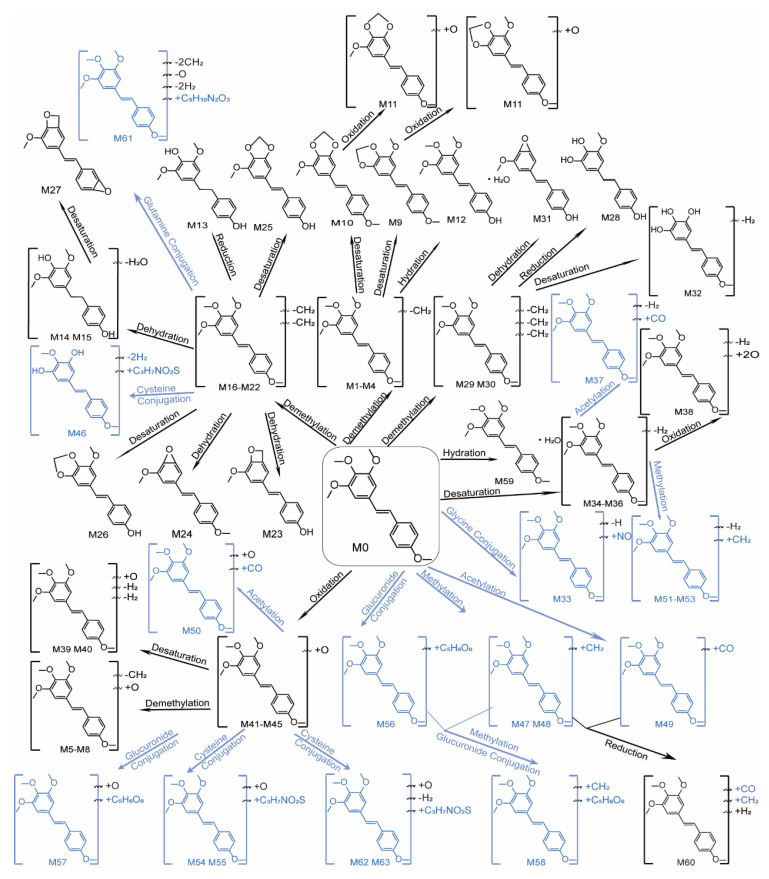
Possible representative structures of proposed metabolic pathways for DMU-212 in Apc^Min/+^ mice (Phase I metabolites are black; Phase II metabolites are blue).

**Figure 6:**
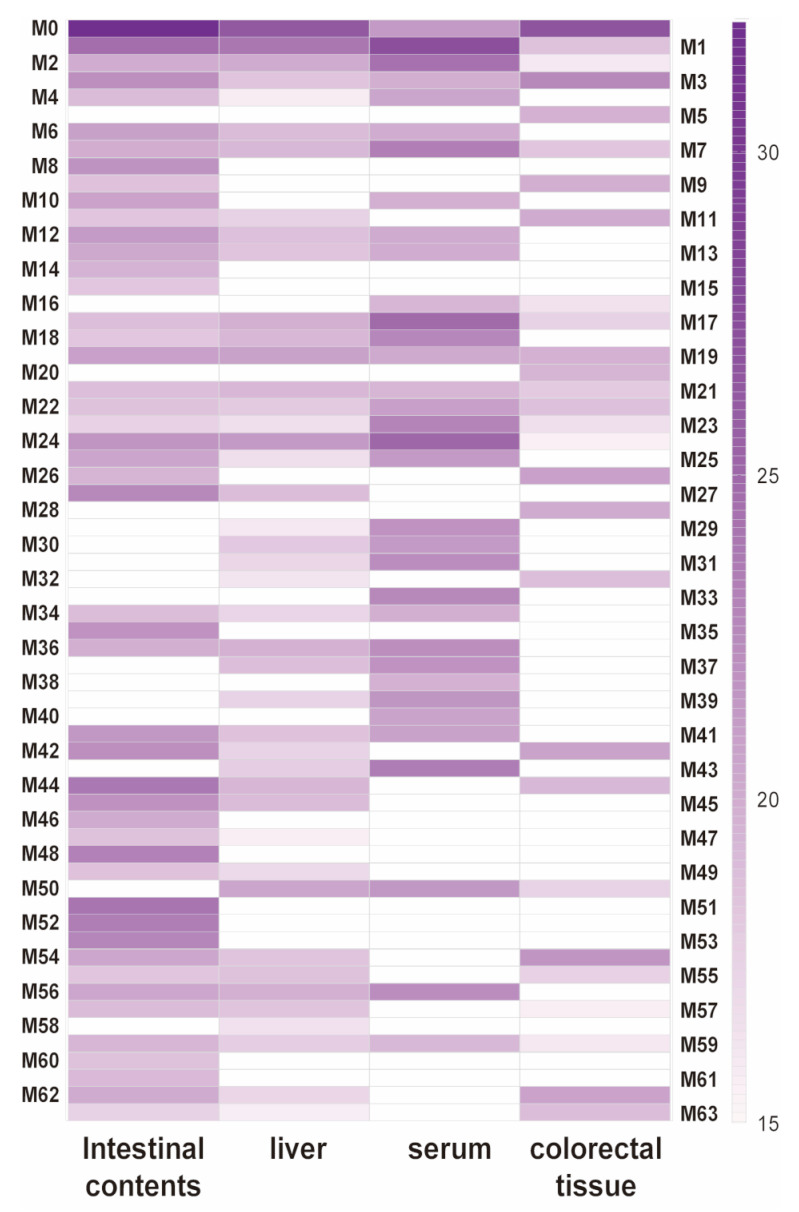
The relative percentage of 63 metabolites of DMU-212 in the Apc^Min/+^ mice’s serum, liver, colorectal tissues and intestinal contents (blank means that the samples do not contain the compound).

Summarizing the metabolic pathways (Figure 5) of the 63 DMU-212 metabolites extracted and identified in the blood, liver, colorectal tissues and intestinal contents of the Apc^Min/+^ mice administrated with DMU-212. A total of 43 Phase I metabolites of DMU-212 were identified, most of which were produced through demethylation, oxidation, desaturation, dehydration, reduction and hydration. A total of 20 Phase II metabolites were identified. Among them, most were methylation, acetylation, glucuronide, cysteine, glycine and glutamine conjugation. As shown in Figure 4B, the methylated and demethylated metabolic reaction in the Apc^Min/+^ mice’s intestinal contents, the demethylated, dehydrated and demethylated reaction in the liver, and the demethylated, oxidized and demethylated reaction in the serum and colorectal tissue, were the primary metabolic steps according to the relative percentage of metabolite type. Additionally, the prototype DMU-212 was found in all serum, liver, colorectal tissues and intestinal contents. The parent drug that was most abundant in the liver, colorectal tissues and intestinal contents was not also the most abundant in the serum, and demethylated metabolite M1 was detected as the most abundant metabolite found in the serum. Except for the parent drug, metabolites M1, M51 and M44 were more abundant in the intestinal contents of the Apc^Min/+^ mice, and metabolites M1, M24 and M19 were most abundant in the liver; metabolites M3, M54 and M26 were most abundant in the colorectal tissues. The data are presented in Figure 6.

### 2.5. Molecular Docking

**Figure 7:**
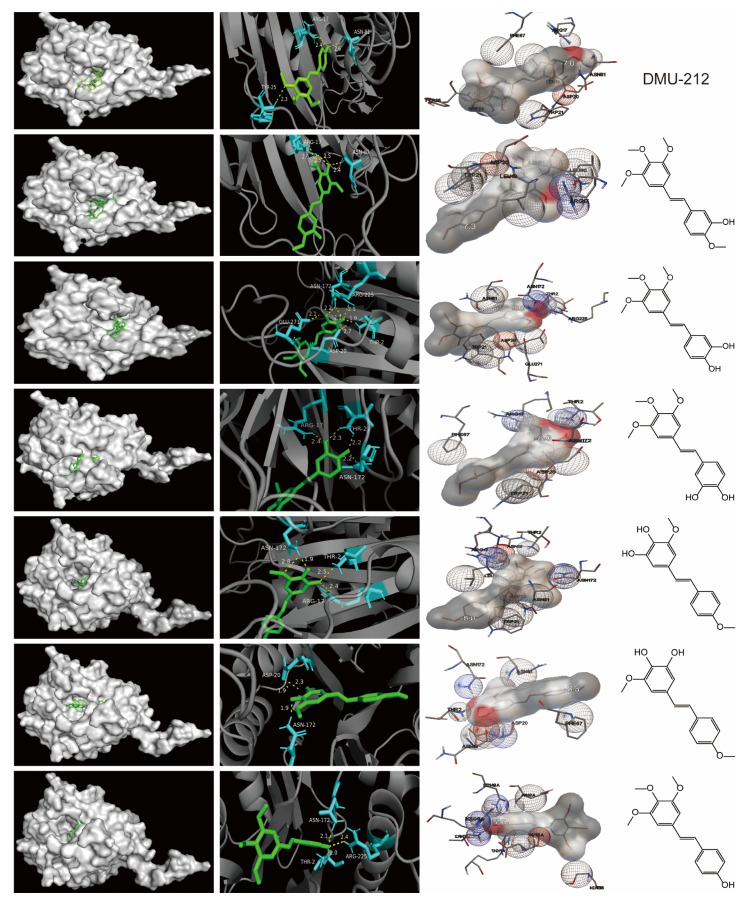
Molecular docking patterns (Green sticks represent ligands, blue sticks represent the protein residues, the dashed yellow lines represent hydrogen bonds between ligands and the protein. gray spheres: van der Waals force, blue spheres: electrostatic interaction force, red spheres: intermolecular repulsion.).

Microbial bile salt hydrolases contribute to the pathogenesis of colon cancer through the bile acid signaling pathway [9]. BSHs may be a potential target for colorectal cancer therapy. Based on the results described above, combined with the literature, prototype, demethylated metabolites, oxidized metabolites, demethylated and oxidized metabolites, which have higher relative percentages in colorectal tissue, were molecularly docked using AutoDock Vina, as shown in Table S3. The absolute value of the binding energy indicates the affinity of the ligands with the target and the conformational stability. The absolute value greater than 4.25 kcal/mol, 5.0 kcal/mol, and 7.0 kcal/mol indicates a certain, good and strong binding activity, respectively [10]. The molecular docking results showed that the binding ability of all DMU-212 metabolites to BSHs was stronger, and the absolute values of the binding energy of each active component were stronger than that of DMU-212 (7.0 kcal/mol). Clusters having a relatively higher absolute value of binding energy were selected and the specific binding patterns were processed and optimized (Figure 7).

## 3. Discussion

Identifying drug metabolites can be crucial to finding potential drug targets, developing safe drug treatments in clinics, and even rationally modifying drugs [11]. On one hand, for the present drug metabolite identification studies, the components in biological samples and their metabolites are obtained with healthy or disease-model animal-based approaches [12,13]. We believe that a core approach to obtaining the active ingredients that serve a therapeutic role can be based on the effectiveness of the medicine [14]. Only in this case, where a specific medication proves to be efficacious, are the components in biological samples analyzed to clarify the material basis and mechanism of action for the drug’s efficacy. On the other hand, in order to identify metabolites and to better understand the structure of drugs, high-quality tandem mass spectra are essential [15]. Without previous experience and prediction of metabolic pathways, identifying low abundant metabolite ions in in vivo samples with a complicated biological matrix presents a substantial challenge [8]. In this study, UHPLC-Q/Orbitrap/LTQ MS combining AcquireX^TM^, a novel data-dependent acquisition workflow, was used to analyze DMU-212 metabolic profiles in vivo based on the effectiveness of the medicine. Through the AcquireX^TM^ data acquisition workflow, background subtraction and method updating were performed in real-time. Hence, only the MS^2^ of potential metabolites were triggered using the updated method without any user intervention. In total, 63 DMU-212 metabolites were identified after the oral dose of 240 mg/kg for 3 weeks in CRA spontaneous model Apc^Min/+^ mice, including 48 metabolites in the intestinal contents, 48 metabolites in the liver, 34 metabolites in the serum, and 28 metabolites in the colorectal tissues, centering on the further reaction of the prototype, demethylated DMU-212 and oxidized DMU-212.

As we know, before oral drugs can be used to enter the blood circulation in the body, they must first be digested in the gastrointestinal tract in order to be absorbed in a suitable form [16]. As a result, the potential metabolic role of gut microbiota on drug metabolism is gaining increasing attention [17,18,19,20]. Our research found that the gut microbiota may be an important site of DMU-212 metabolism in vivo, and DMU-212 mainly undergoes rapid Phase I, such as demethylation, desaturation, dehydration and oxidation, and Phase II, including methylation metabolism, in the gut microbiota. There is increasing evidence that the gut microbiota affects drug metabolism by altering the structural and functional properties of drugs, which is primarily mediated by unique enzymes encoded within the microbiome [21,22,23]. That DMU-212 is being metabolized by gut microbiota may also have something to do with the fact that the gut microbiota contain a wide range of enzymes. Firmicutes and Bacteroidetes were the dominating taxa in the gut microbiota, which have many enzymes, such as oxidase, reductase, and esterase, allowing for many catalytic processes such as oxidation (hydration, hydrogenation, hydroxylation, methylation, oxygenation), and reduction (dehydration, dehydroxylation, demethylation) [24,25]. After being processed by gut microbes, most drugs, including DMU-212, are reabsorbed by the intestinal epithelium and then enter the enterohepatic circulation. In addition to being the main metabolic organ, the liver is also the main site of drug metabolism. As we expected, 48 metabolites were observed in the Apc^Min/+^ mice’s liver, which inferred that the liver possessed high metabolic activity for DMU-212. In the liver, CYP450 plays an important role in drug metabolism [26]. A previous study demonstrated that via CYP1A1, DMU-212 may be demethylated to DMU-218 and oxidized to DMU-214, and the biological activity of the parent compound may be dependent on its conversion to DMU-214 and the level of this enzyme [27]. In contrast, our results showed that DMU-212 not only included demethylation and oxidation in the Apc^Min/+^ mice’s liver but was also involved in many other reactions including Phase I, such as desaturation and dehydration, and even Phase II reactions, such as acetylation, glucuronide and cysteine conjugation, which were responsible for the identification of abundant DMU-212 metabolites. In general, the drug is metabolized in the liver and then distributed into the blood. During previous research, DMU-212 was rapidly cleared from the blood within an hour after administration, and a small amount of the prototype was detected [7]. In this paper, in the serum of the Apc^Min/+^ mice orally administered with DMU-212, the parent drug was also low, which indicated that DMU-212 was metabolized by intestinal bacteria or was absorbed into the blood and underwent hepatic first-pass elimination, followed by Phase I and II metabolism to be quickly transformed into the other metabolites, especially the demethylated, dehydrated and oxidized metabolites involved in Phase I, and the acetylated, glycine/glucuronide-bound metabolites involved in Phase II observed in this paper.

Medicines enter the bloodstream and are delivered to the target tissue where they exert their therapeutic impact. Colorectal tissue is the target tissue in which DMU-212 might prevent malignancy or delay its onset. In our study, in addition to the most abundant parent drug detected in the Apc^Min/+^ mice’s colorectal tissue, demethylated, desaturated and oxidized metabolites were also found. In addition, the oxidized and cysteine-bound metabolite M54 was also a major Phase II metabolite and found at a relatively high level in colorectal tissue. Phase II reactions convert compounds to more water-soluble and often less active or toxic derivatives to increase excretion [28]; therefore, metabolite M54 may not be the pharmacologically active constituent. Bile acid metabolism associated with gut microbiota may contribute to the pathogenesis of colon cancer. In addition, our recent findings underline the regulatory roles of DMU-212 in dysregulated gut microbiota and BA metabolism to prevent CRA in Apc^Min/+^ mice (our unpublished data). Microbial BSHs, which initiate bile acid metabolism, are highly related to CRC, these enzymes have been considered a promising target in the manipulation of gut microbiota to benefit human health [9]. Therefore, we further explored the possible molecular interactions of the metabolites obtained in the colorectal tissue with BSHs using molecular docking experiments. Molecular docking showed that the target BSHs had a strong binding activity with the main metabolites. In addition to the reported five in vivo metabolites [7], several novel demethylated and/or oxidized metabolites have been characterized from the Apc^Min/+^ mice’s colorectal tissue, which may affect bile acid metabolism to prevent CRA by acting on the BSHs’ target. These provided a hint that DMU-212 metabolic products may be the pharmacodynamic material basis of DMU-212 to prevent CRA. However, it is still necessary to conduct further experiments to confirm this speculation.

## 4. Materials and Methods

### 4.1. Chemicals and Reagents

The resveratrol analogue DMU-212 was synthesized according to the reference method [29]. Its purity (>98%) (Figures S1 and S2) and structure were determined using nuclear magnetic resonance spectroscopy (Figure S3, Table S1). Methanol, formic acid and acetonitrile (HPLC grade) were purchased from Fisher Scientific (Fisher Scientific, Waltham, MA, USA).

### 4.2. Animal Experiments

All of the experiments were approved by the Animal Care Welfare Committee of the Heilongjiang University of Chinese Medicine. In total, 20 Apc^Min/+^ mice (males aged 4 w) were obtained from GemPharmatech Co. Ltd. (Nanjing, China) and housed under a standard 12-h light-dark cycle at 25 ± 2 °C and 60 ± 5% humidity with free access to water and a high-fat diet (Research Diets, D12492; 60% fat by calories). At 7 weeks of age, the Apc^Min/+^ mice were randomly assigned to the vehicle-treated Apc^Min/+^ (MOD) and DMU-212-treated Apc^Min/+^ groups (DMU) (10 mice per group). The MOD group was orally administrated 0.5 % sodium carboxymethyl cellulose (CMC-Na) per day. The DMU group was orally administrated 240 mg/kg of DMU-212 per day. Drug treatment was performed once daily for 28 successive days. The efficacy of DMU-212 prevention for CRA was evaluated using blood feces, colon length, spleen index, adenoma number, histopathology and inflammatory cytokines, and CRA-related protein expression in colonic tissues. The data is being published in detail in a different publication.

### 4.3. Collection and Preparation of Biological Samples

Blood, liver, colorectal tissues and intestinal contents samples were collected at 1 h after the last administration. The serum was obtained through the centrifugation of blood in a refrigerated centrifuge at 3000 rpm (4 °C). A 300 μL volume of the serum sample was added to 900 µL of methanol and vortexed for 1.0 min to precipitate the protein and the sample was centrifuged at 12,000 rpm and 4 °C for 10 min. The supernatants were then concentrated using a speed vacuum concentrator and the residue was redissolved using 100 µL of methanol and centrifuged at 12,000 rpm for 10 min. In total, 0.1 g intestinal contents were collected and resuspended in 1 mL of methanol followed by centrifugation (12,000× *g* rpm, 4 °C, 10 min). The supernatants were dried in vacuo and resuspended in 200 μL of methanol and centrifuged at 12,000 rpm for 10 min. A total of 0.2 g of the mice’s liver tissue and colorectal tissues were weighed, respectively, and homogenized in 1 mL of methanol, and then centrifuged at 12,000 g for 10 min at 4 °C. The supernatant was concentrated in vacuo and redissolved in 200 µL of methanol, and centrifuged at 4 °C at 12,000× *g* rpm for 10 min. Finally, the supernatants collected from the four kinds of samples were passed through a 0.22 μm membrane filter and a 2 μL sample was injected into the UHPLC-Q/Orbitrap/LTQ MS system for analysis.

### 4.4. UHPLC-Q/Orbitrap/LTQ MS Analysis

The liquid chromatographic separations were performed on a Vanquish (Thermo Fisher Scientific, Waltham, MA, USA) system hyphenated with a Q/Orbitrap/LTQ mass spectrometer system (Thermo Fisher Scientific). As a stationary phase, a Waters Corporation ACQUITY UPLCTM BEH C18 column (100 mm × 2.1 mm, i.d., 1.7 microns) was used. Mobile phase A was 0.1% formic acid in water and mobile phase B was 0.1% formic acid in acetonitrile. The gradient was established as follows: 0–2 min, 1–10% B; 2–4 min, 10–40% B; 4–5 min, 40–65% B; 5–12 min, 65–100% B; 12–13 min, 100% B; 13–14 min, 100–1% B; and 14–17 min, 1% B. The flow rate was 0.3 mL/min and the column temperature was set at 35 °C.

A heated electrospray ionization interface, working in positive mode, was used in the mass spectrometer. The parameters were the following: spray voltage of 3.5 kV; sheath gas of 40 arb; and auxiliary gas of 15 arb. The ion transfer tube and the vaporizer temperature were at 350 °C. The scanning mode was full MS/DD-MS^2^ based on the AcquireX^TM^ intelligent data acquisition technology (Thermo Fisher Scientific), with an Orbitrap resolution of 120,000 and a mass range of *m*/*z* 50–1200.

### 4.5. Data Processing Software

The Xcalibur 4.0 workstation software (Thermo Fisher Scientific) was used for raw data recording and processing. Using Compound Discoverer 3.3 (Thermo Fisher Scientific), post-processing of the data was performed to extract the metabolite-related datasets based on the structural correlation between the drug and its metabolites. A maximum tolerance of 5 ppm was set for the mass error. Using blank samples, the workflow subtracted the chemical backgrounds, aligned the retention times, and found the expected compounds and metabolites. Fragment ion search (FISH) scoring was used, and each annotation was then manually evaluated based on the HCD, DDA spectra, molecular formula, and FISH coverage.

### 4.6. Molecular Docking

The 3D structure of bile salt hydrolases (BSHs) was downloaded from the RCSB PDB (https://www.rcsb.org/, accessed on 18 April 2023). The PubChem database (https://pubchem.ncbi.nlm.nih.gov/, accessed on 18 April 2023) was used to download the SDF format files of the 2D structure of DMU-212. The structures of another core active component were made with ChemDraw (http://www.perkinelmer.com/category/chemdraw, accessed on 18 April 2023). Molecular docking with the main active components of the main active ingredients and BSHs was performed using PyMoL 2.3.0 and AutoDock Vina 18. The binding activity was assessed using binding energy.

## 5. Conclusions

In this paper, the metabolic profiles of DMU-212 in Apc^Min/+^ mice’s serum, liver, colorectal tissues and intestinal contents were systematically and comprehensively investigated based on the effectiveness of the medicine. A total of 63 DMU-212 metabolites were determined and summarized through quick, sensitive and accurate UHPLC-Q/Orbitrap/LTQ MS combined with the data processing software “Compound Discoverer 3.3” and the AcquireX^TM^ data acquisition workflow. In addition, further verification of the representative active metabolites was employed using molecular docking analysis. The Phase I metabolites of DMU-212 were mainly produced via demethylation, oxidation, desaturation, dehydration, reduction and hydration, while the major Phase II metabolites were methylation, acetylation, glucuronide, cysteine, glycine and glutamine conjugation products. As a result of this study, a simple method for studying drugs’ metabolism in vivo is provided, as is scientific and reliable support for a complete understanding of DMU-212’s metabolism and transformation. In addition, as a reference for the further development of new drugs, this is also important for a deeper understanding of the active constituents of DMU-212 and its action mechanisms for CRA prevention.

## Data Availability

The data presented in this study are available in the main article.

## Supplementary Materials

The following supporting information can be downloaded at: https://www.mdpi.com/article/10.3390/molecules28093828/s1, Figure S1: UV absorption spectrum for DMU-212 (200-400nm); Figure S2: The chromatogram of DMU-212 was analyzed using HPLC-PDA; Figure S3: Structural characterization of DMU-212 using NMR spectral analysis: 1 H NMR spectrum (A), the magnified 1 H NMR spectrum (B), 13C NMR spectrum (C), the magnified 13C NMR spectrum (D); Figure S4: MS^2^ spectra of DMU-212 and its metabolites (Green indicates fragment-matched). Table S1: The 1 H and 13 C NMR data attribution of DMU-212; Table S2. Summary of DMU-212 Metabolites in the Apc^Min/+^ mice blood, liver, colorectal tissues and intestinal contents; Table S3 Docking results of BSHs and core active components.
