# Supplementary material for: In Vivo Metabolite Profiling of DMU-212 in ApcMin/+ Mice Using UHPLC-Q/Orbitrap/LTQ MS
Source: Molecules. 2023 Apr 30;28(9):3828. doi: 10.3390/molecules28093828 (PMC10180202; doi:10.3390/molecules28093828)
Supplement: Supplementary file 1 [file molecules-28-03828-s001.zip › molecules-2335833-supplementary.pdf]

SUPPORTING INFORMATION

In Vivo Metabolite Profiling of DMU-212 in Apc $^{Min/+}$ mice UHPLC-Q/Orbitrap/LTQ MS

Jing Li[1],Xinghua Li[1], Xiaohang Zhou[1,2], Le Yang[3], Hui Sun[1]*, Ling Kong[1], Guangli Yan[1], Ying Han[1], Xijun Wang[1,2,3]*,

1 National Chinmedomics Research Center, National TCM Key Laboratory of Serum Pharmacochemistry, Metabolomics Laboratory, Department of Pharmaceutical Analysis, Heilongjiang University of Chinese Medicine, Heping Road 24, Harbin 150040, China

2 State Key Laboratory of Quality Research in Chinese Medicine, Macau University of Science and Technology, Avenida Wai Long, Taipa, Macau 999078, China

3 State Key Laboratory of Dampness Syndrome, The Second Affiliated Hospital Guangzhou University of Chinese Medicine, Dade Road 111, Guangzhou 510006, China

---

* Corresponding author:

Prof. Hui Sun

National Chinmedomics Research Center, National TCM Key Laboratory of Serum Pharmacochemistry, Department of Pharmaceutical Analysis, Heilongjiang University of Chinese Medicine, Heping Road 24, Harbin 150040, China.

Tel. & Fax +86-451-87260818

Email: Sunhui7045@sina.com

Prof. Wang Xi-Jun

National Chinmedomics Research Center, National TCM Key Laboratory of Serum Pharmacochemistry, Metabolomics Laboratory, Heilongjiang University of Chinese Medicine, Heping Road 24, Harbin 150040, China.

Tel. & Fax +86-451-82110818

Email: xijunw@sina.com

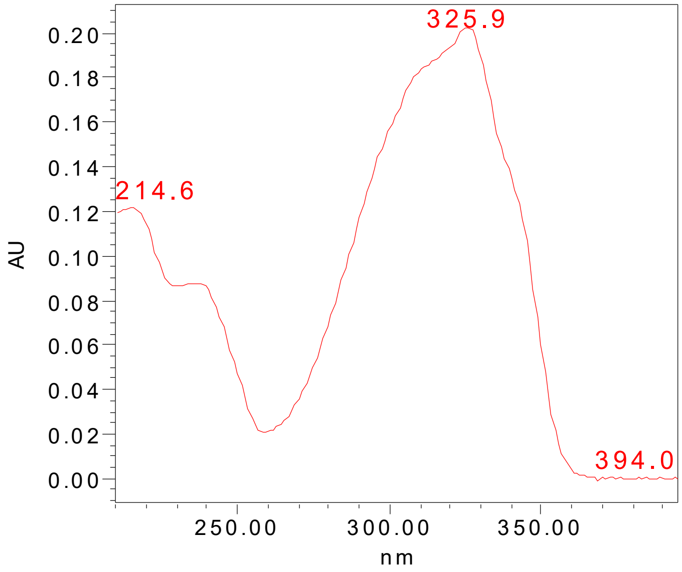

Figure S1 UV absorption spectrum for DMU-212 (200-400nm).

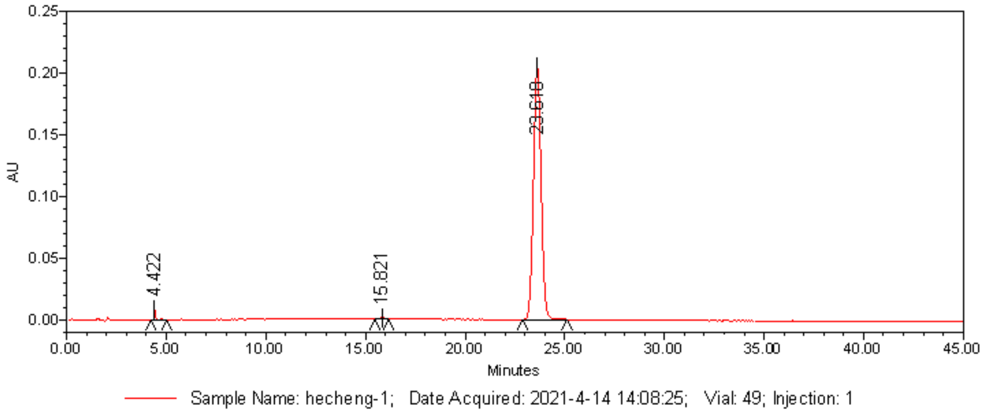

Peak Results

	SampleName	Name	Retention Time (min)	Area	% Area	Height	Amount	Units
1	hecheng-1		4.422	55443	0.99	8615		
2	hecheng-1		15.821	22384	0.40	1284		
3	hecheng-1		23.618	5508253	98.61	204465		

Figure S2 The chromatogram of DMU-212 was analyzed using HPLC-PDA.

Table S1 The 1 H and 13 C NMR data attribution of DMU-212.

	^{1}H NMR(500MHz)	^{13}C NMR
Position	δ_H	δ_C
1		114.18
2	6.74(2H,s)	126.58
3		130.03
3-CH_3O	3.94(6H,s)	56.13
4		137.69
4-CH_3O	3.89(3H,s)	60.98
5		129.29
5-CH_3O	3.94(6H,s)	56.13
6	6.74(2H,s)	126.58
7	7.48(1H,d,J=10 Hz)	159.32
8	7.46(1H,d,J=10 Hz)	153.41
1’		103.35
2’	6.93(1H,dd,J=10, 5 Hz)	127.77
3’	6.91(1H,dd,J=10, 5 Hz)	127.65
4’		133.46
4’-CH_3O	3.85(3H,s)	55.34
5’	6.92(1H,dd,J=10, 5 Hz)	127.65
6’	6.94(1H,dd,J=10, 5 Hz)	127.77

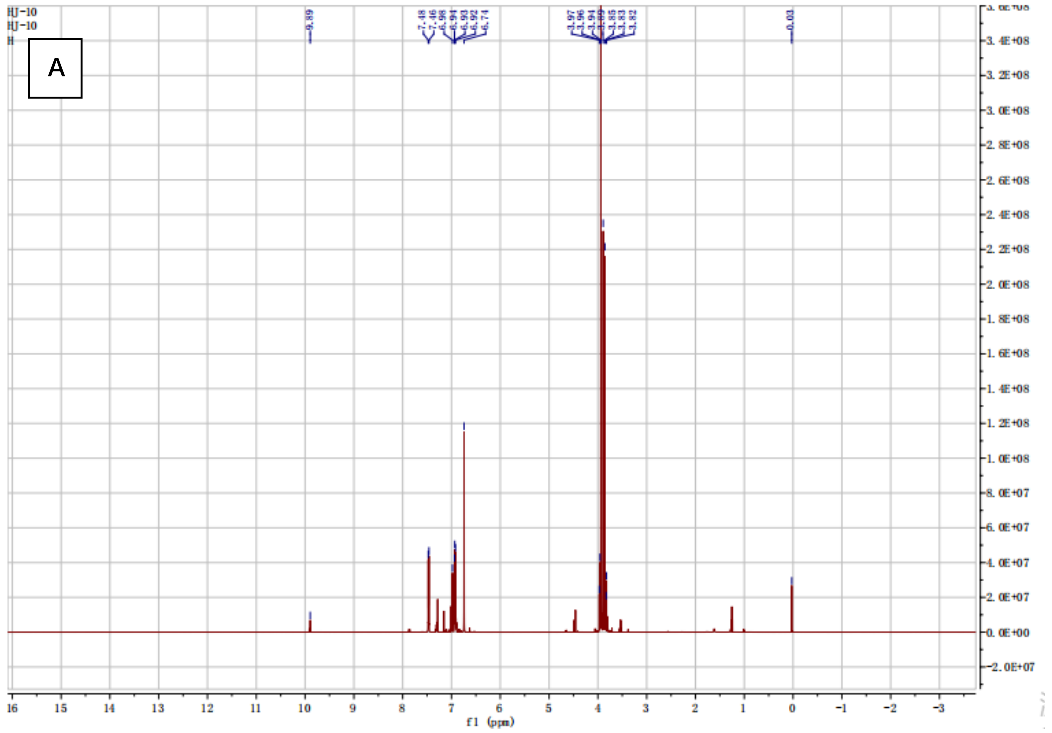
HJ-10
HJ-10
H
A
9.89
7.48
7.46
6.98
6.94
6.93
6.92
6.74
3.97
3.96
3.94
3.85
3.83
3.82
0.03
f1 (ppm)

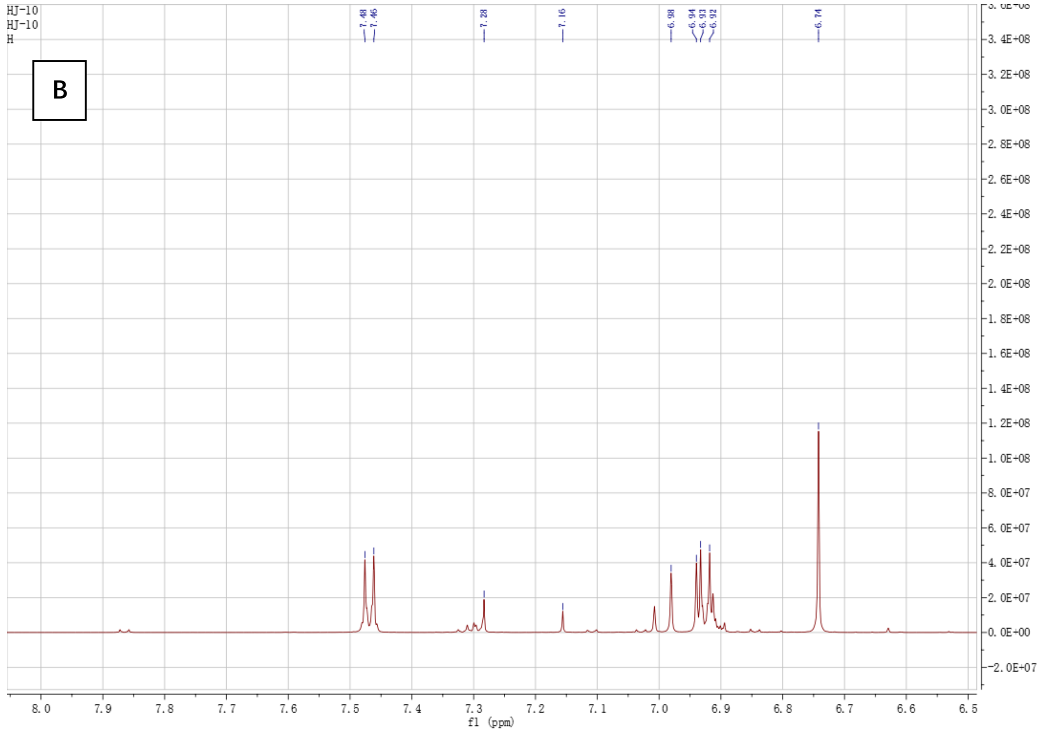
HJ-10
HJ-10
H
B
7.48
7.46
7.28
7.16
6.98
6.94
6.93
6.92
6.74
f1 (ppm)

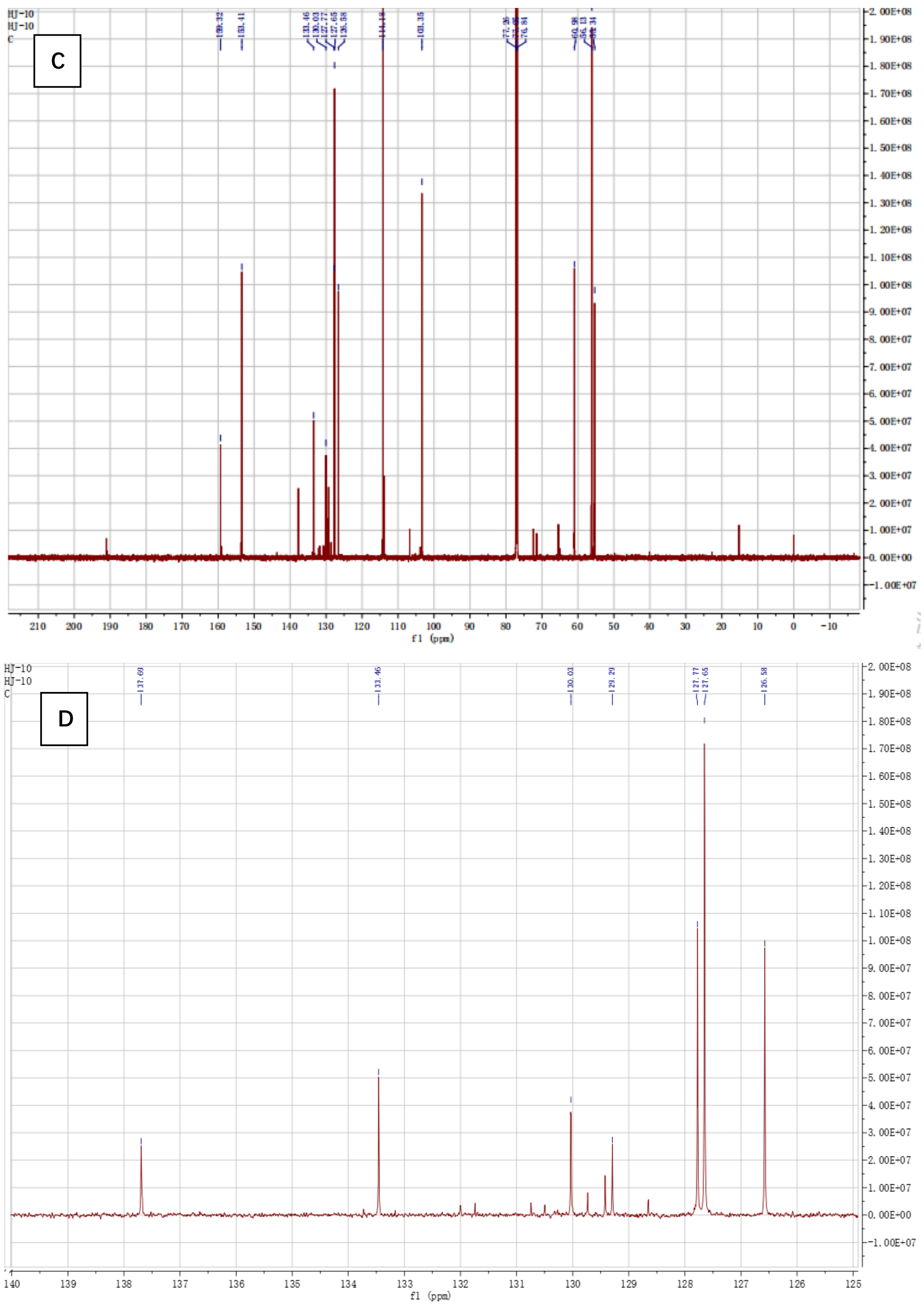

Figure S3 Structural characterization of DMU-212 by NMR spectral analysis: 1 H NMR spectrum (A), the magnified 1 H NMR spectrum (B),13C NMR spectrum (C), the magnified 13C NMR spectrum (D)

Table S2. Summary of DMU-212 Metabolites in the Apc $^{Min/+}$ mice blood, liver, colorectal tissues and intestinal contents.

Peak NO.	RT /min	Molecular formula	Neutral mass /Da	Observed m/z	Mass error /ppm	Transformations	Composition Change	Intestinal Contents	Liver	Serum	Colorectal Tissues	Type
M0	7.454	$C_{18} H_{20} O_4$	300.13587	301.14314	-0.97	Parent		+	+	+	+	I
M1	6.127	$C_{17} H_{18} O_4$	286.12026	287.12754	-0.85	Demethylation	-(C H_2)	+	+	+	+	I
M2	6.003	$C_{17} H_{18} O_4$	286.12024	287.12752	-0.93	Demethylation	-(C H_2)	+	+	+	+	I
M3	6.878	$C_{17} H_{18} O_4$	286.12022	287.12749	-1.02	Demethylation	-(C H_2)	+	+	+	+	I
M4	5.336	$C_{17} H_{18} O_4$	286.12027	287.12754	-0.85	Demethylation	-(C H_2)	+	+	+		I
M5	6.349	$C_{17} H_{18} O_5$	302.11525	303.12253	-0.58	Demethylation, Oxidation	-(C H_2) +(O)				+	I
M6	4.781	$C_{17} H_{18} O_5$	302.11516	303.12243	-0.88	Demethylation, Oxidation	-(C H_2) +(O)	+	+	+		I
M7	5.923	$C_{17} H_{18} O_5$	302.11518	303.12246	-0.81	Demethylation, Oxidation	-(C H_2) +(O)	+	+	+	+	I
M8	6.784	$C_{17} H_{18} O_5$	302.11514	303.12241	-0.94	Demethylation, Oxidation	-(C H_2) +(O)	+				I
M9	6.848	$C_{17} H_{16} O_4$	284.10462	285.11190	-0.84	Demethylation, Desaturation	-(C H_4)	+			+	I
M10	5.291	$C_{17} H_{16} O_4$	284.10464	285.11192	-0.76	Demethylation, Desaturation	-(C H_4)	+	+	+		I
M11	6.272	$C_{17} H_{16} O_5$	300.09956	301.10684	-0.72	Demethylation, Desaturation, Oxidation	-(C H_4) +(O)	+	+		+	I
M12	5.554	$C_{17} H_{20} O_5$	304.13082	305.13809	-0.85	Demethylation, Hydration	-(C) +(O)	+	+	+		I
M13	5.533	$C_{16} H_{18} O_4$	274.12023	275.12750	-1.03	Demethylation, Reduction	-(C_2 H_2)	+	+	+		I
M14	6.66	$C_{16} H_{16} O_3$	256.10981	257.11708	-0.54	Dehydration, Demethylation, Reduction	-(C_2 H_4 O)	+				I

M15	6.292	$C_{16}H_{16}O_3$	256.10971	257.11699	-0.9	Dehydration, Demethylation, Reduction	$-(C_2H_4O)$	+				I
M16	6.877	$C_{16}H_{16}O_4$	272.10457	273.11184	-1.07	Demethylation	$-(C_2H_4)$		+	+	+	I
M17	6.745	$C_{16}H_{16}O_4$	272.10458	273.11186	-1.02	Demethylation	$-(C_2H_4)$	+	+	+	+	I
M18	5.718	$C_{16}H_{16}O_4$	272.10460	273.11188	-0.93	Demethylation	$-(C_2H_4)$	+	+	+		I
M19	5.512	$C_{16}H_{16}O_4$	272.10464	273.11192	-0.8	Demethylation	$-(C_2H_4)$	+	+	+	+	I
M20	6.524	$C_{16}H_{16}O_4$	272.10476	273.11203	-0.38	Demethylation	$-(C_2H_4)$				+	I
M21	6.095	$C_{16}H_{16}O_4$	272.10460	273.11188	-0.95	Demethylation	$-(C_2H_4)$	+	+	+	+	I
M22	6.219	$C_{16}H_{16}O_4$	272.10460	273.11188	-0.94	Demethylation	$-(C_2H_4)$	+	+	+	+	I
M23	6.331	$C_{16}H_{14}O_3$	254.09407	255.10135	-0.87	Dehydration, Demethylation	$-(C_2H_6O)$	+	+	+	+	I
M24	6.144	$C_{16}H_{14}O_3$	254.09406	255.10133	-0.94	Dehydration, Demethylation	$-(C_2H_6O)$	+	+	+	+	I
M25	5.333	$C_{16}H_{14}O_4$	270.08898	271.09625	-0.86	Desaturation, Demethylation	$-(C_2H_6)$	+	+	+		I
M26	6.523	$C_{16}H_{14}O_4$	270.08905	271.09633	-0.59	Desaturation, Demethylation	$-(C_2H_6)$	+			+	I
M27	7.455	$C_{16}H_{12}O_3$	252.07843	253.08571	-0.83	Dehydration, Desaturation, Demethylation	$-(C_2H_8O)$	+	+			I
M28	5.743	$C_{15}H_{16}O_4$	260.10462	261.11189	-0.93	Demethylation, Reduction	$-(C_3H_4)$				+	I
M29	6.191	$C_{15}H_{14}O_4$	258.08896	259.09624	-0.95	Demethylation	$-(C_3H_6)$		+	+		I
M30	5.637	$C_{15}H_{14}O_4$	258.08898	259.09625	-0.9	Demethylation	$-(C_3H_6)$		+	+		I
M31	5.691	$C_{15}H_{12}O_3$	240.07849	241.08577	-0.64	Dehydration, Demethylation	$-(C_3H_8O)$		+	+		I
M32	5.799	$C_{15}H_{12}O_4$	256.07335	257.08063	-0.82	Desaturation, Demethylation	$-(C_3H_8)$		+		+	I
M33	6.325	$C_{18}H_{19}NO_5$	329.12607	330.13335	-0.76	Demethylation, Glycine	-(H) +(N O)			+		II

						Conjugation						
M34	5.389	$C_{18}H_{18}O_4$	298.12034	299.12762	-0.56	Desaturation	$-(H_2)$	+	+	+		I
M35	7.94	$C_{18}H_{18}O_4$	298.12034	299.12761	-0.58	Desaturation	$-(H_2)$	+				I
M36	6.12	$C_{18}H_{18}O_4$	298.12027	299.12754	-0.81	Desaturation	$-(H_2)$	+	+	+		I
M37	6.599	$C_{19}H_{18}O_5$	326.11516	327.12243	-0.81	Desaturation, Acetylation	$-(H_2) +(CO)$		+	+		II
M38	6.047	$C_{18}H_{18}O_6$	330.11000	331.11727	-1.04	Desaturation, Oxidation, Oxidation	$-(H_2) +(O_2)$		+	+		I
M39	6.048	$C_{18}H_{16}O_5$	312.09951	313.10679	-0.83	Desaturation, Desaturation, Oxidation	$-(H_4) +(O)$		+	+		I
M40	6.571	$C_{18}H_{16}O_5$	312.09938	313.10665	-1.27	Desaturation, Desaturation, Oxidation	$-(H_4) +(O)$			+		I
M41	5.295	$C_{18}H_{20}O_5$	316.13082	317.13809	-0.82	Oxidation	$+(O)$	+	+	+		I
M42	6.63	$C_{18}H_{20}O_5$	316.13087	317.13815	-0.64	Oxidation	$+(O)$	+	+		+	I
M43	5.957	$C_{18}H_{20}O_5$	316.13085	317.13812	-0.72	Oxidation	$+(O)$		+	+		I
M44	7.45	$C_{18}H_{20}O_5$	316.13084	317.13812	-0.73	Oxidation	$+(O)$	+	+		+	I
M45	6.295	$C_{18}H_{20}O_5$	316.13082	317.13810	-0.81	Oxidation	$+(O)$	+	+			I
M46	3.867	$C_{19}H_{21}NO_6S$	391.10872	392.11600	-0.6	Demethylation, Cysteine Conjugation	$+(CHNO_2S)$	+	+			II
M47	5.257	$C_{19}H_{22}O_4$	314.15151	315.15878	-0.96	Methylation	$+(CH_2)$	+	+			II
M48	8.077	$C_{19}H_{22}O_4$	314.15152	315.15880	-0.92	Methylation	$+(CH_2)$	+	+			II
M49	5.873	$C_{19}H_{20}O_5$	328.13083	329.13811	-0.74	Acetylation	$+(CO)$	+	+			II

M50	6.599	$C_{19}H_{20}O_6$	344.12568	345.13296	-0.88	Oxidation, Acetylation	$+(CO_2)$		+	+	+	II
M51	7.46	$C_{19}H_{20}O_4$	312.13591	313.14319	-0.79	Desaturation, Methylation	$+(C)$	+				II
M52	8.936	$C_{19}H_{20}O_4$	312.13589	313.14317	-0.85	Desaturation, Methylation	$+(C)$	+				II
M53	7.912	$C_{19}H_{20}O_4$	312.13591	313.14319	-0.8	Desaturation, Methylation	$+(C)$	+				II
M54	5.442	$C_{21}H_{27}NO_7S$	437.15030	438.15757	-1.2	Oxidation, Cysteine Conjugation	$+(C_3H_7NO_3S)$	+	+		+	II
M55	5.532	$C_{21}H_{27}NO_7S$	437.15056	438.15783	-0.61	Oxidation, Cysteine Conjugation	$+(C_3H_7NO_3S)$	+	+		+	II
M56	6.112	$C_{23}H_{26}O_{10}$	462.15206	463.15934	-1.16	Glucuronide Conjugation	$+(C_5H_6O_6)$	+	+	+		II
M57	5.785	$C_{23}H_{26}O_{11}$	478.14701	479.15429	-1.04	Oxidation, Glucuronide Conjugation	$+(C_5H_6O_7)$	+	+		+	II
M58	6.302	$C_{24}H_{28}O_{10}$	476.16752	477.17475	-1.54	Glucuronide Conjugation, Methylation	$+(C_6H_8O_6)$		+			II
M59	5.846	$C_{18}H_{22}O_5$	318.14646	319.15374	-0.82	Hydration	$+(H_2O)$	+	+	+	+	I
M60	5.374	$C_{20}H_{24}O_5$	344.16203	345.16931	-1	Reduction, Acetylation	$+(C_2H_4O)$	+				II
M61	5.002	$C_{21}H_{22}N_2O_6$	398.14756	399.15484	-0.56	Desaturation, Demethylation, Glutamine Conjugation	$+(C_3H_2N_2O_2)$	+				II
M62	5.564	$C_{21}H_{25}NO_7S$	435.13468	436.14196	-1.12	Oxidation, Cysteine Conjugation	$+(C_3H_5NO_3S)$	+	+		+	II
M63	5.647	$C_{21}H_{25}NO_7S$	435.13477	436.14205	-0.92	Oxidation, Cysteine Conjugation	$+(C_3H_5NO_3S)$	+	+		+	II

Table S3 Docking results of BSHs and core active components.

NO.	Ligands	Binding Energy (kcal/mol)
0	DMU-212	-7
1	DMU-212+O-1	-7.3
2	DMU-212+O-1-CH_2-1	-8
3	DMU-212+O-1-CH_2-2	-7.5
4	DMU-212+O-1-CH_2-3	-7.4
5	DMU-212+O-1-CH_2-4	-7.6
6	DMU-212+O-2	-7.6
7	DMU-212+O-2-CH_2-1	-8
8	DMU-212+O-2-CH_2-2	-7.6
9	DMU-212+O-2-CH_2-3	-7.5
10	DMU-212+O-2-CH_2-4	-7.5
11	DMU-212+O-3	-7.1
12	DMU-212+O-3-CH_2-1	-7.8
13	DMU-212+O-3-CH_2-2	-7.2
14	DMU-212+O-3-CH_2-3	-7.1
15	DMU-212+O-3-CH_2-4	-7.3
16	DMU-212+O-4	-7.2
17	DMU-212+O-4-CH_2-1	-7.7
18	DMU-212+O-4-CH_2-2	-7.2
19	DMU-212+O-4-CH_2-3	-7.1
20	DMU-212+O-4-CH_2-4	-7.5
21	DMU-212+O-5	-7.1
22	DMU-212+O-5-CH_2-1	-7.2
23	DMU-212+O-5-CH_2-2	-7.1
24	DMU-212+O-5-CH_2-3	-7
25	DMU-212+O-5-CH_2-4	-7.4
26	DMU-212+O-6	-7.2
27	DMU-212+O-6-CH_2-1	-7.4
28	DMU-212+O-6-CH_2-2	-7.2
29	DMU-212+O-6-CH_2-3	-7.2
30	DMU-212+O-6-CH_2-4	-7.2
31	DMU-212-C_2H_4-1	-7.5
32	DMU-212-C_2H_4-2	-7.4
33	DMU-212-C_2H_4-3	-7.5
34	DMU-212-C_2H_4-4	-8
35	DMU-212-C_2H_4-5	-7.5
36	DMU-212-C_2H_4-6	-8.3
37	DMU-212-CH_2-1	-7.5
38	DMU-212-CH_2-2	-7.2
39	DMU-212-CH_2-3	-7.2
40	DMU-212-CH_2-4	-7.2

M0

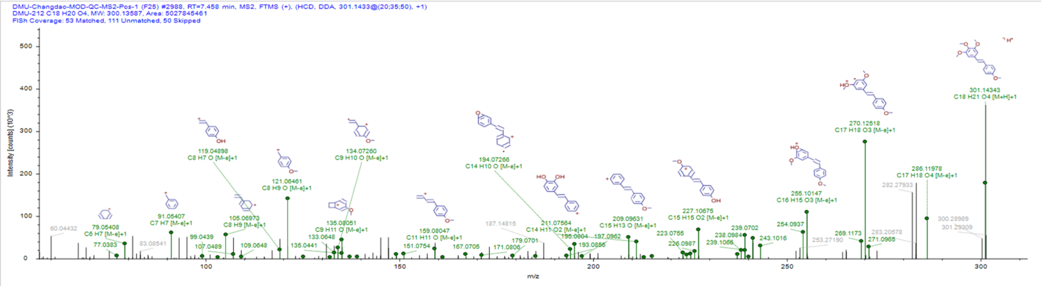

M1-4

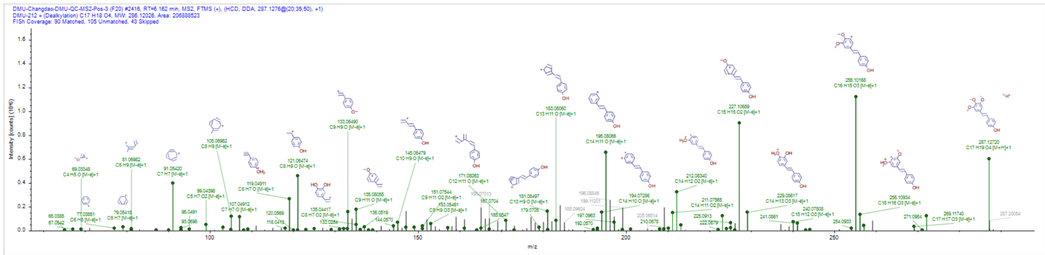

M16-22

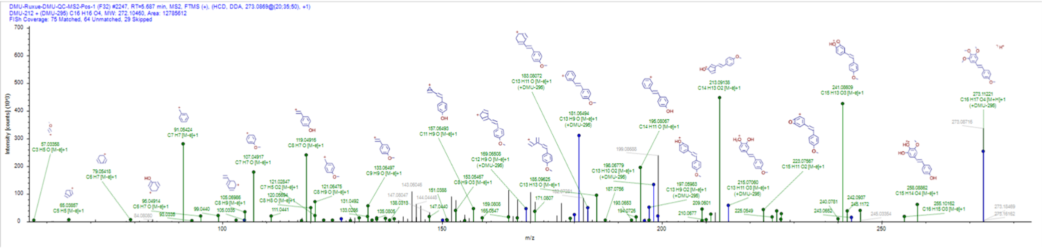

M29-30

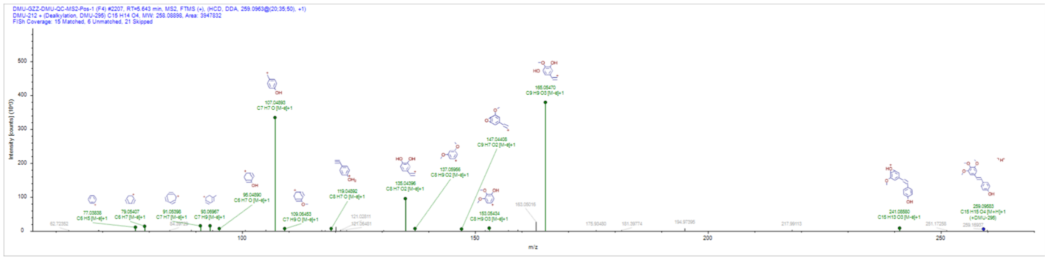

M34-36

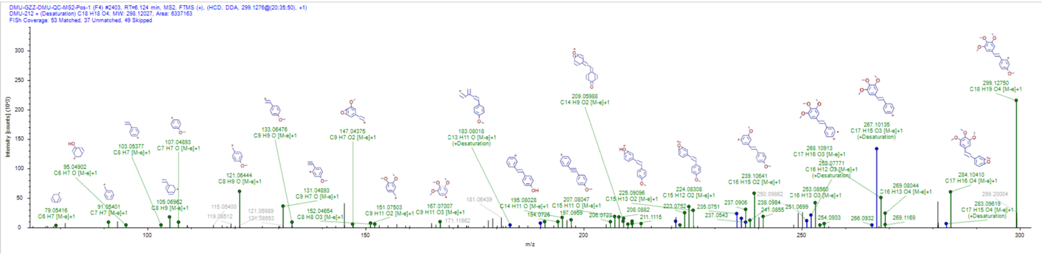

M9-10

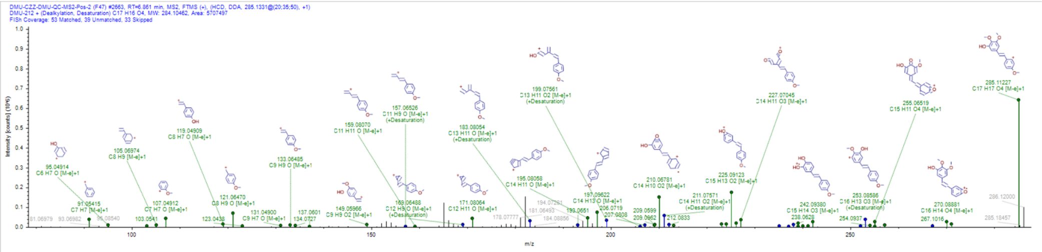

M25-26

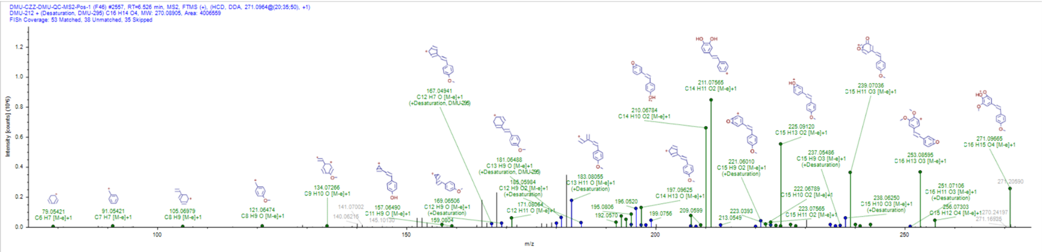

M32

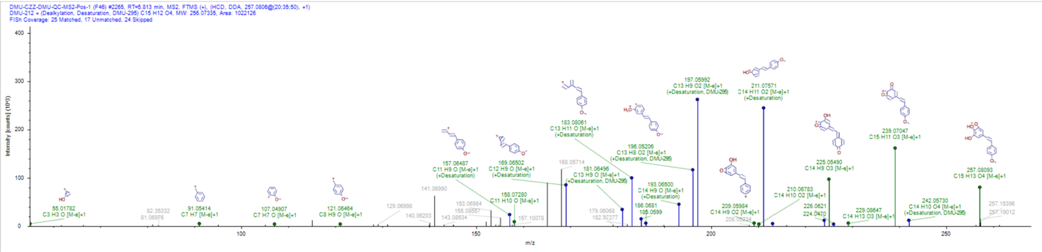

M41-45

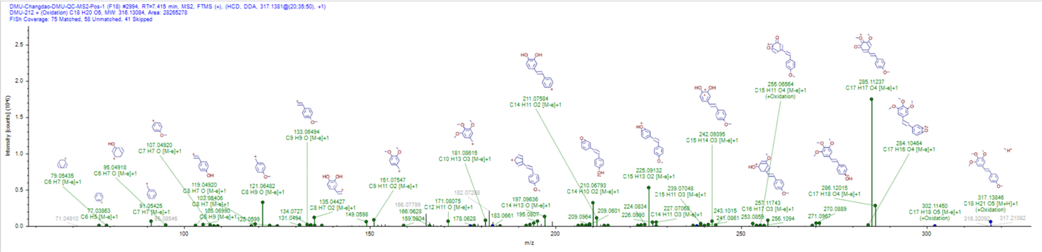

M39-40

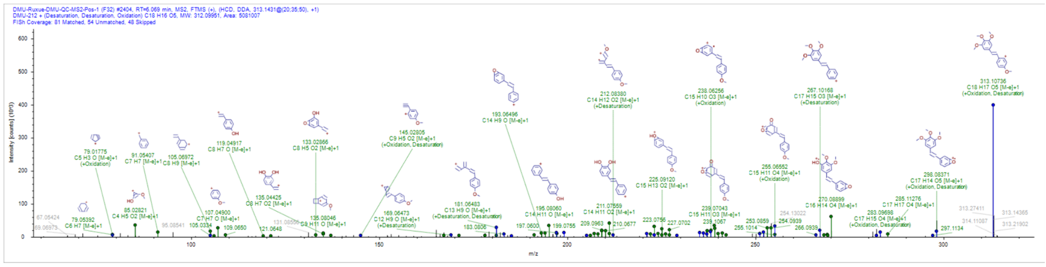

M38

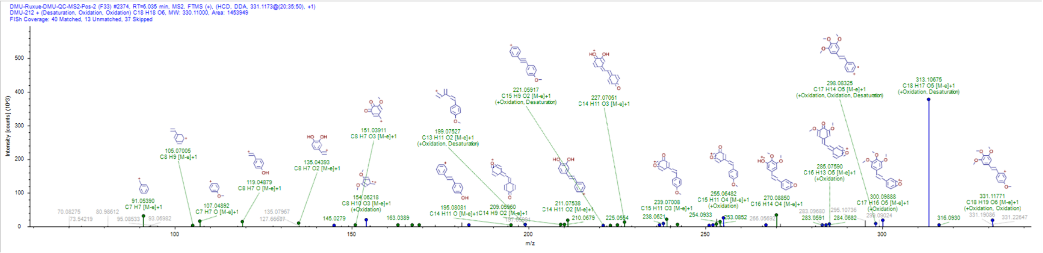

M23-24

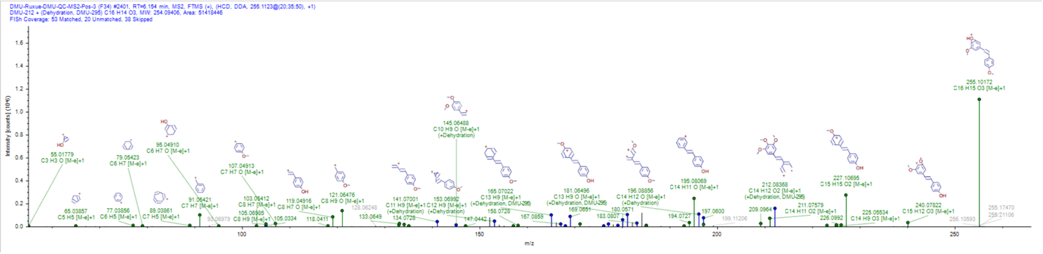

M31

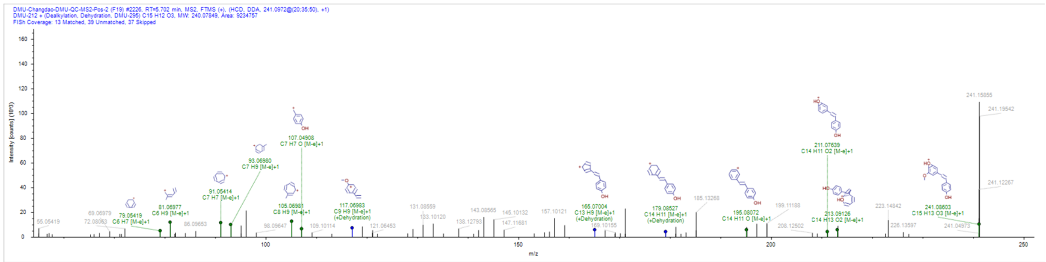

M14-15

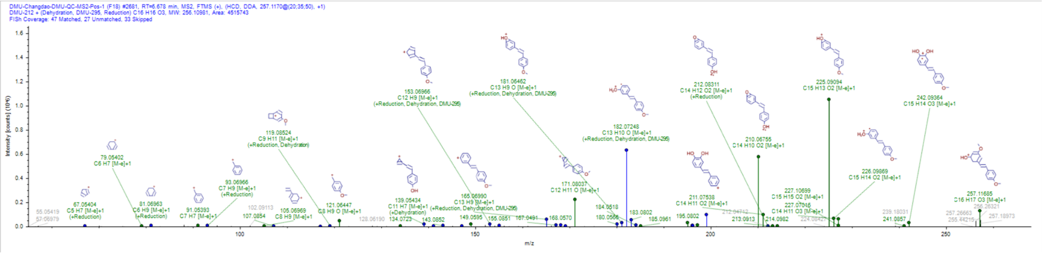

M27

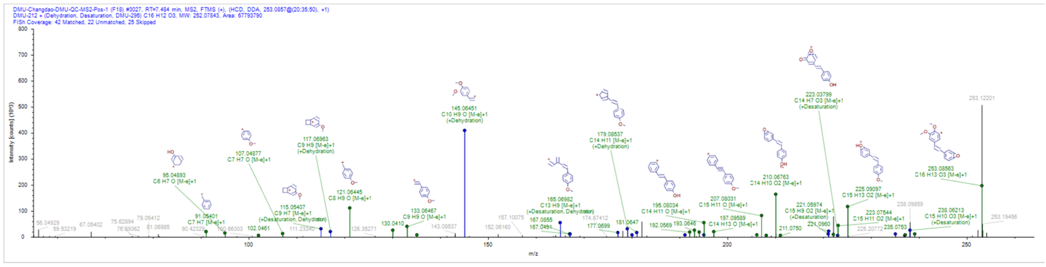

M5-8

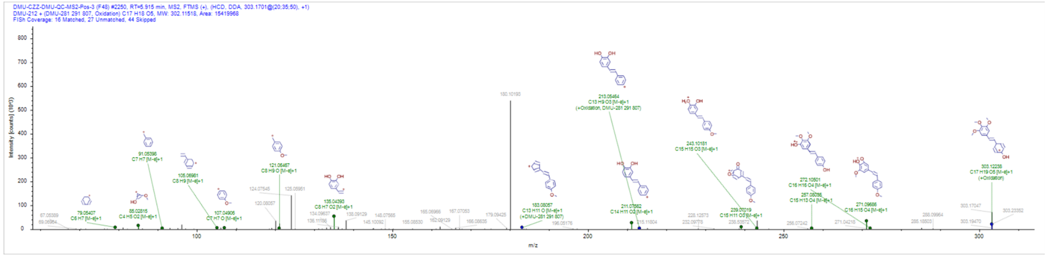

M13

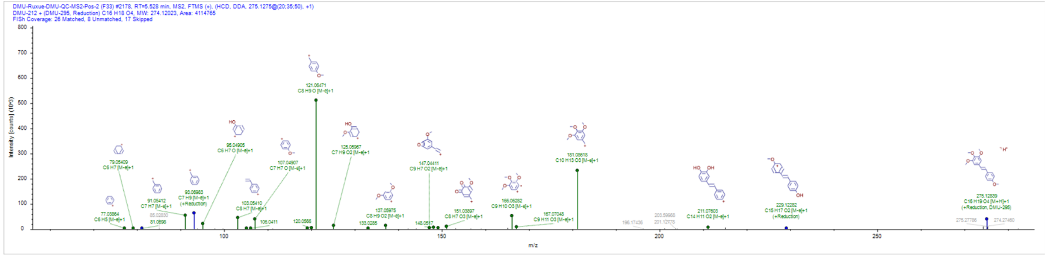

M28

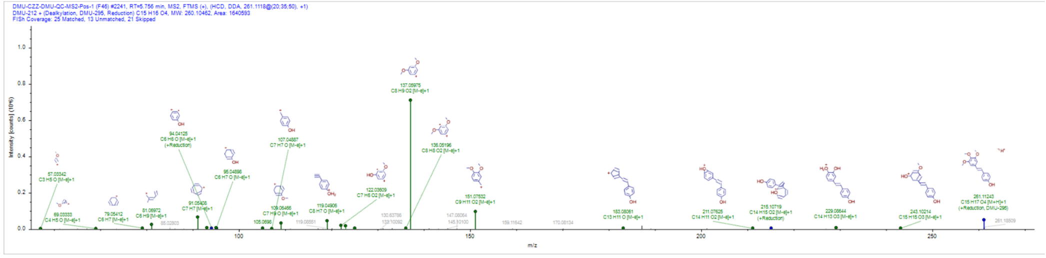

M11

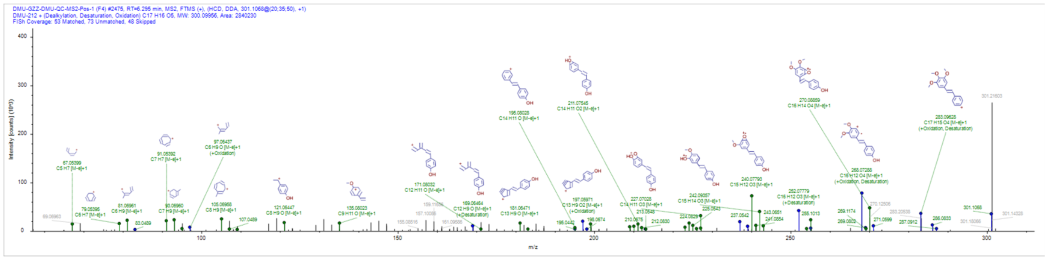

M12

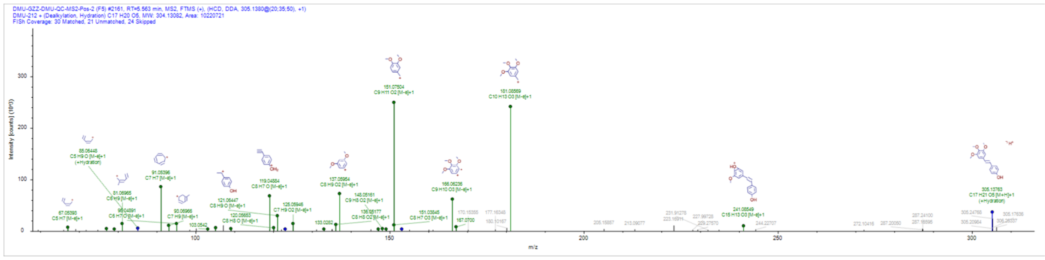

M59

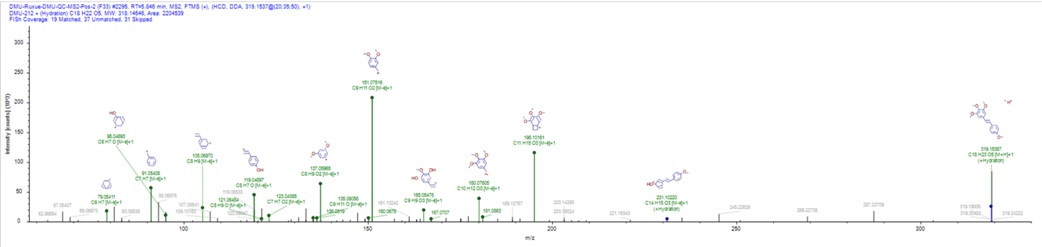

M56

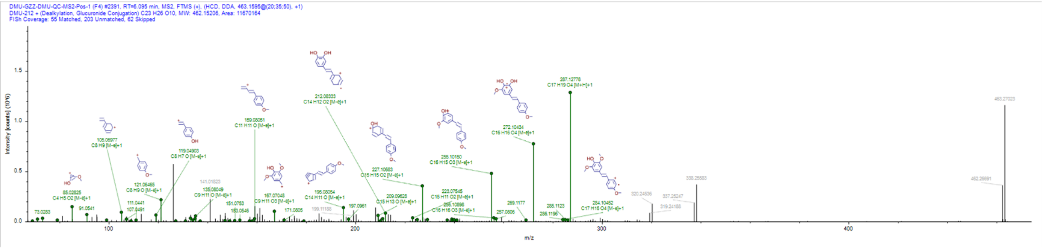

M57

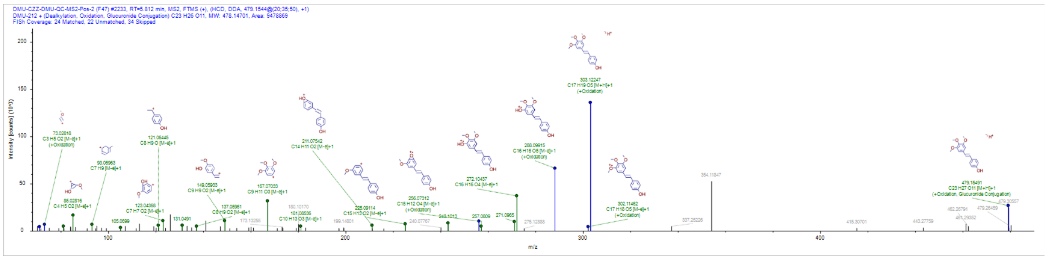

M47-48

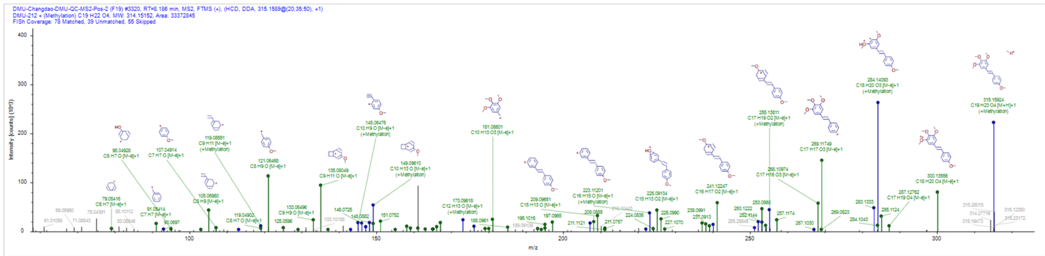

M51-53

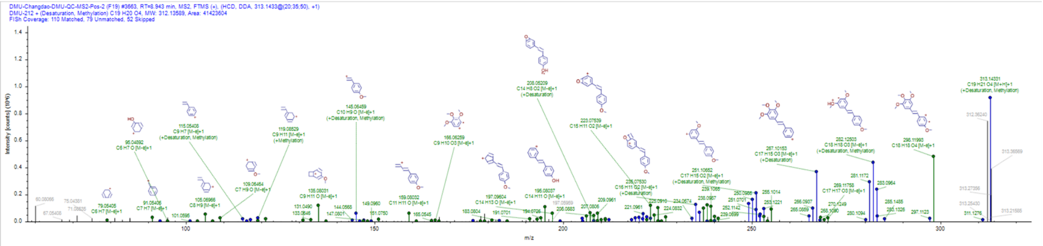

M58

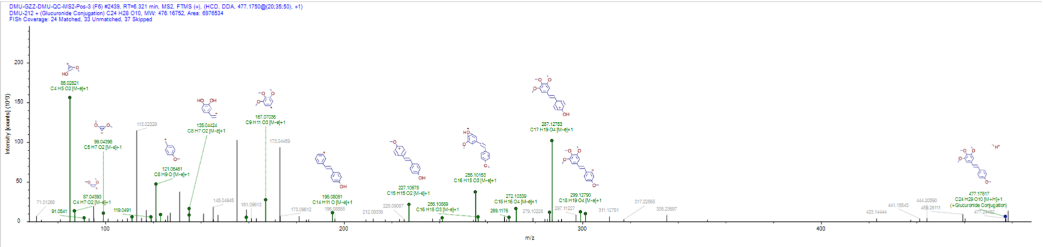

M49

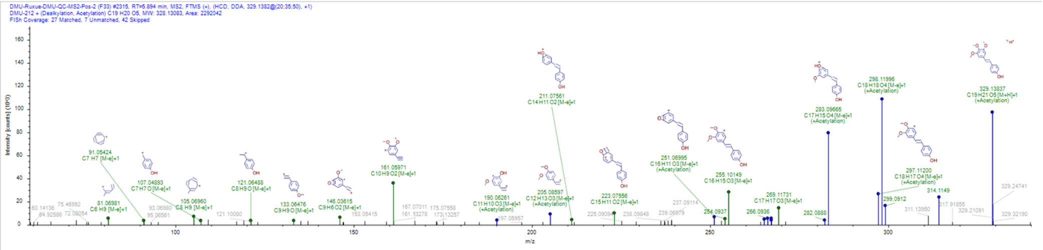

M50

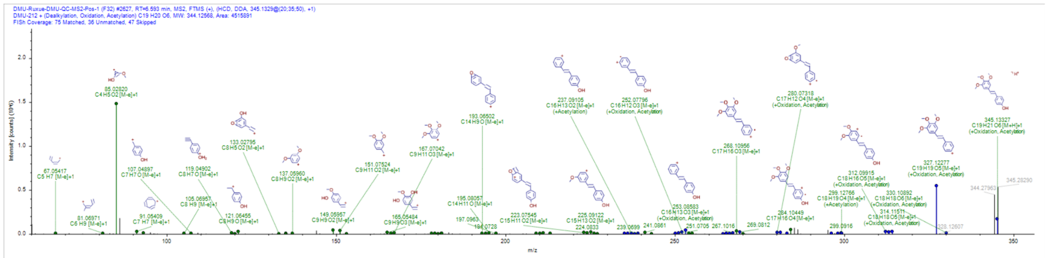

M37

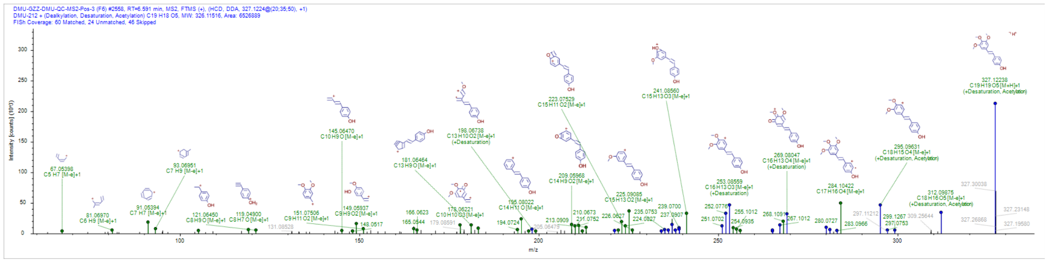

M60

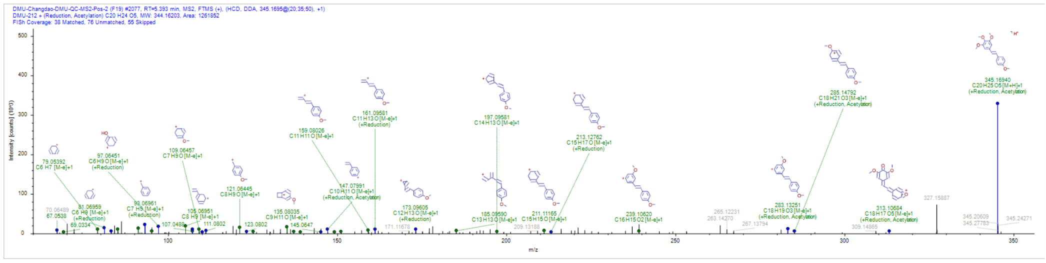

M54-55

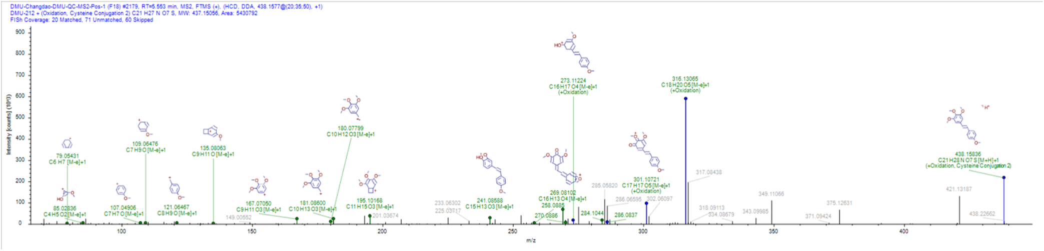

M62-63

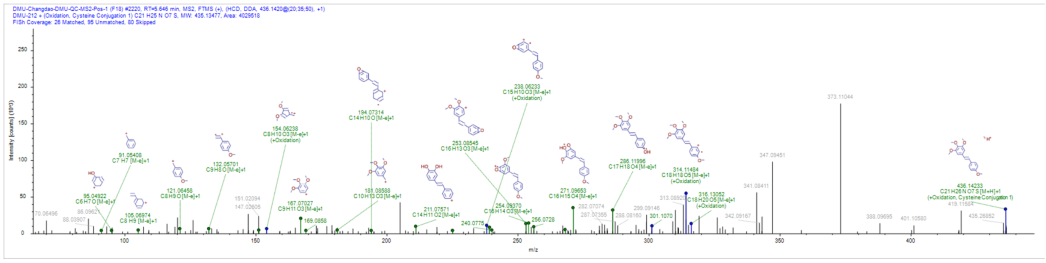

M46

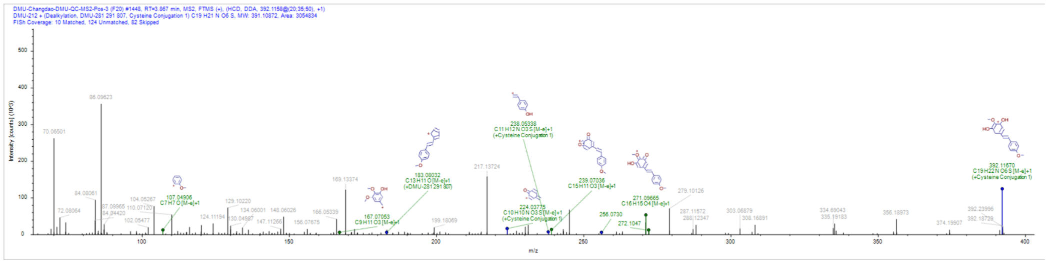

M33

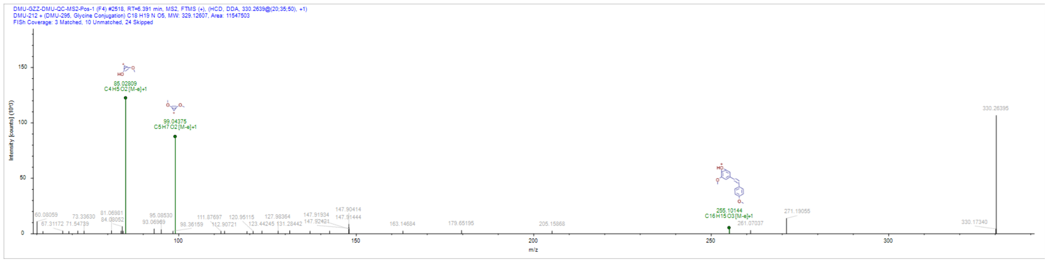

M61

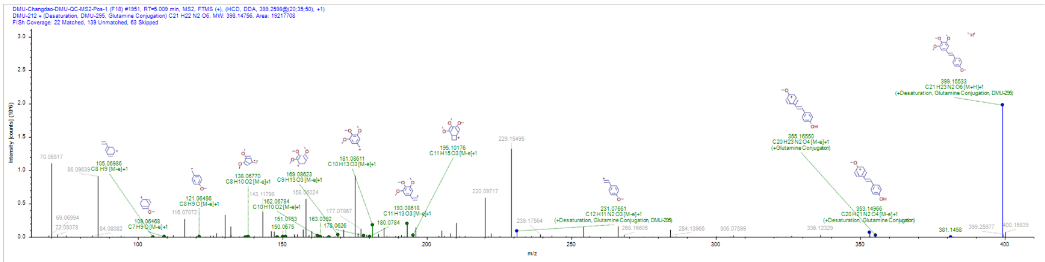

Figure S4 MS^2 spectra of DMU-212 and metabolites (Green indicate fragment-matched)
